# Goat milk protein digestibility in relation to intestinal function

**DOI:** 10.1093/ajcn/nqaa400

**Published:** 2021-03-01

**Authors:** Sindhu Kashyap, Nirupama Shivakumar, Veerasamy Sejian, Nicolaas E P Deutz, Thomas Preston, Sheshshayee Sreeman, Sarita Devi, Anura V Kurpad

**Affiliations:** Division of Nutrition, St. John's Research Institute, St. John's National Academy of Health Sciences, Bangalore, India; Division of Nutrition, St. John's Research Institute, St. John's National Academy of Health Sciences, Bangalore, India; ICAR—National Institute of Animal Nutrition and Physiology, Adugodi, Bangalore, India; Center for Translational Research in Aging and Longevity, Department of Health and Kinesiology, Texas A&M University, College Station, TX, USA; Scottish Universities Environmental Research Centre, East Kilbride, Scotland, UK; Department of Crop Physiology, University of Agricultural Sciences, Bangalore, India; Division of Nutrition, St. John's Research Institute, St. John's National Academy of Health Sciences, Bangalore, India; Division of Nutrition, St. John's Research Institute, St. John's National Academy of Health Sciences, Bangalore, India; Department of Physiology, St. John's Medical College, St. John's National Academy of Health Sciences, Bangalore, India

**Keywords:** milk protein, intrinsic labeling, dual-isotope tracer technique, lactulose to mannitol ratio, amino acid absorption

## Abstract

**Background:**

Milk is an important high-quality animal protein source in low- and middle-income countries (LMICs). Although the true ileal digestibility and absorption of milk has been shown to be high in French adults, this may be lower in individuals from LMICs who are at risk of environmental enteropathy.

**Objective:**

To determine the true ileal indispensable amino acid (IAA) digestibility of intrinsically labeled goat milk protein in South Indian women of reproductive age (WRA), using the dual-isotope tracer technique, and to measure intestinal absorption of amino acid and inert sugar in the same participants using L-allo-isoleucine and a dual-sugar assay.

**Methods:**

Milk with ^2^H-labeled protein collected from a lactating goat fed intrinsically ^2^H-labeled fodder (maize and cowpea) was spray dried. Labeled milk protein was administered in a plateau feeding protocol to WRA with normal BMI, in whom urinary lactulose and mannitol recovery and the lactulose/mannitol ratio (LMR) were measured, to determine its true ileal IAA digestibility by the dual-isotope tracer technique with a reference U-^13^C–amino acid mixture. A phenylalanine absorption index was calculated from the plasma to meal ratio of ^13^C_9_ phenylalanine within the digestibility protocol. On a separate day, the allo-isoleucine absorption index was estimated from the ratio of plasma allo-isoleucine enrichments after oral ^13^C_6_-^15^N-L- and intravenous ^2^H_10_-L-allo-isoleucine administration.

**Results:**

The means ± SDs of true ileal IAA digestibility of goat milk protein, lactulose and mannitol recovery, LMR, allo-isoleucine and phenylalanine absorption index were 94.0 ± 2.9%, 0.09 ± 0.03%, 7.9 ± 2.3%, 0.012 ± 0.004, 88.4 ± 3.8% and 24.5 ± 1.6%, respectively. The LMR correlated with the allo-isoleucine absorption index (*r*_s_ = –0.93, *P* = 0.008).

**Conclusion:**

The true ileal digestibility of goat milk protein in South Indian WRA with normal intestinal absorptive function and integrity was comparable to earlier estimates in healthy French adults.

## Introduction

Animal source foods (ASFs) have been shown to be beneficial for growth, cognitive performance, physical activity, and health, particularly in children and pregnant women (1–3). Milk is a common ASF in many low- and middle-income countries (LMICs), and a recent analysis of a nationally representative Indian survey underlined the association of household milk consumption, represented by maternal consumption, with a moderate reduction (3%) in stunting of their preschool children (4). Milk protein consumption during pregnancy in South Indian women was also shown to be positively associated with birth weight (5). These observations emphasize the importance of ASF consumption, particularly in India, where the risk of quality-corrected protein inadequacy is high (6).

The quality of an ASF protein is dependent not only on its amino acid score but also on its true ileal indispensable amino acid (IAA) digestibility, which is the combination of protein digestion and IAA absorption at the ileal level (7). Milk is shown to be a high-quality protein with optimum amino acid score and high digestibility in high-income countries (HICs) (8). Although food processing and the food matrix can affect protein digestibility (9), another factor that could limit digestibility is the impairment of intestinal function by environmental enteropathy (EE) mostly prevalent in LMICs (10). Earlier reports suggest lower brush-border enzyme activity and reduced sugar, amino acid, and dipeptide absorption in asymptomatic adults from LMICs compared with their Western counterparts (11, 12). In comparison to US and Jamaican women, healthy South Indian women showed lower intestinal absorptive capacity and functional enterocyte mass, as measured by mannitol absorption (37% and 41% compared with 15%) and citrulline flux (12 and 10 compared with 9 μmol/kg fat-free mass/h), along with a unique gut microbiota with functional correlates (13). Asymptomatic Indians residing in the United Kingdom also showed altered intestinal morphology and greater intestinal permeability compared with the native population (14). The question therefore arises whether milk protein is equally well digested and absorbed in LMICs compared with HICs.

The true ileal IAA digestibility of a protein can be indirectly measured by the minimally invasive dual stable isotope tracer technique (15) by comparing the plasma appearance of individual IAAs from an intrinsically (^2^H) labeled test protein with that of a differently (^13^C) labeled reference protein/free IAAs of known digestibility (15). The technique, when referenced against free IAAs, represents digestion, with an assumption of similar absorption of the test and reference amino acids. Small intestinal permeability and absorption can be assessed by the noninvasive dual-sugar assay with lactulose and mannitol (16). Intestinal amino acid absorption can be measured as an absorption index resulting from the administration of isotopically labeled nonprotein isotopologues of L-amino acids, such as allo-isoleucine, via oral and intravenous routes (17, 18). This study aimed to measure the true ileal digestibility of goat milk protein in healthy South Indian women of reproductive age (WRA) by the dual-isotope tracer technique while also characterizing their intestinal absorption by the allo-isoleucine absorption index and intestinal permeability by the dual-sugar assay.

## Methods

### Intrinsic labeling of goat milk

The intrinsic labeling of goat milk protein (hereafter referred to as milk protein) was performed in 2 stages. In the first stage, a fodder crop for goat feed was intrinsically labeled with deuterium oxide (^2^H_2_O, 99.9%; Sercon Ltd), and in the second stage, the ^2^H-labeled fodder was administered to a lactating goat and her milk collected.

Fodder crops, maize (*Zea mays*; var. African black) and cowpea (*Vigna unguiculata*; var. KM 5), were grown in the rainy (kharif) season of 2018 at the University of Agricultural Sciences, Bengaluru, India. A primed pulse labeling as previously described was followed with modifications in the gravimetric irrigation protocol, where the field capacity of the soil was maintained at 100% and 70% for maize and cowpea, respectively, until harvest and the ^2^H_2_O dosing (19). Based on a pilot study to ensure sufficient enrichment of fodder for subsequent adequate labeling of milk protein for a human digestibility study, the ^2^H_2_O pulsed dosing was initiated after the 50% flowering stage with a bolus of 400 mL (35% ^2^H_2_O, w/w) on the first day, followed by 100-mL pulses of 7.5% ^2^H_2_O (w/w) on days 3, 5, 7, and 9 for both crops. An extra 100-mL pulse of 7.5% was administered on day 11 for the maize crop. The plants were harvested 10 d after the last pulse to ensure maximal uptake of residual ^2^H_2_O in the soil, shredded, air dried, and stored for feed.

The Institutional Animal Ethical Committee of St. John's Medical College approved the goat milk intrinsic labeling experiment and handling procedures. A lactating doe (*Capra aegagrus hircus*; common name, *Beetal*; 4 y old, 44-kg weight) and its kid were housed in the Experimental Livestock Unit (ELU) of the National Institute of Animal Nutrition and Physiology (NIANP), Bengaluru, India. The doe was dewormed, vaccinated against enterotoxaemia, and acclimatized to the ELU and the feed (unlabeled dried maize and cowpea fodder, with concentrate mixture prepared by the NIANP) for 45 d before the actual labeling protocol began. For the intrinsic labeling protocol of milk, the dried ^2^H-labeled fodder was substituted into the feed administered to the doe for 17 d. The amount provided was 750 g/d of ^2^H-labeled maize and 160 g/d of ^2^H-labeled cowpea with 500 g/d of unlabeled protein concentrate mixture. The quantity was determined based on the feed consumed by the doe during the acclimatization period and adjusted to provide 60% protein from the labeled fodder and the remaining from the concentrate mixture. The feed administered met the daily protein and energy requirements of a lactating goat in accordance with the National Research Council (20). The amount of leftover feed was quantified each day, ranging from 117 to 493 g and 0 to 21 g of maize and cowpea, respectively. Water was provided ad libitum throughout the study duration.

During this period, the kid was weaned from doe to cow milk to ensure that all the ^2^H-labeled milk was available for the human protein digestibility study. The doe was milked twice daily (morning and evening), and the collected milk was pasteurized immediately at 72^⁰^C for 1 min, then cooled to room temperature and stored in a –20^⁰^C freezer, until it was taken for spray drying. The milk was collected from 2 d before until the 21st day of the labeled fodder feeding protocol. Milk from days 4 to 20 was pooled and spray dried (Bowen Engineering; inlet temperature, 150^⁰^C; outlet temperature, 90^⁰^C) at the Central Food Technological Research Institute, Mysuru, India. The spray-dried milk powder was stored at 4^⁰^C for subsequent human experiments.

### Human studies

All participants were recruited from the staff population of St. John's Research Institute, Bengaluru, India. Healthy nonpregnant and nonlactating women (*n*  = 7) with a normal BMI (between 18.5 and 25 kg/m^2^), aged between 20 to 35 y, were included in the study. Participants who were nonsmokers, without any food allergies, and reporting no medical or surgical illness within 3 mo of the study were selected. Medical history and clinical examination were performed to rule out history of gastrointestinal symptoms, antibiotic and iron supplement intake within 4 wk of the study, nonsteroidal anti-inflammatory drug use, and alcohol consumption in the past 1 wk. All experiments were conducted during the proliferative or secretory phase of their menstrual cycle. Informed written consent was obtained from the participants. The study protocol was approved by the Institutional Ethical Review Board of St. John's Medical College Hospital. Participant screening and enrollment details are provided in **Supplementary Figure 1**.

### Measurement of intestinal permeability, allo-isoleucine absorption, and milk protein digestibility

Along with the measurement of milk protein digestibility, intestinal function was characterized by the evaluation of intestinal permeability by a dual-sugar test and the measurement of allo-isoleucine absorption. While the noninvasive dual-sugar test was performed first, the order of the invasive (intravenous administration and blood sampling) allo-isoleucine absorption and milk protein digestibility was randomly allocated using a research randomizer tool. In total, there were 3 separate experiment days conducted within 2 wk.

### Dual-sugar absorption test

**FIGURE 1:**
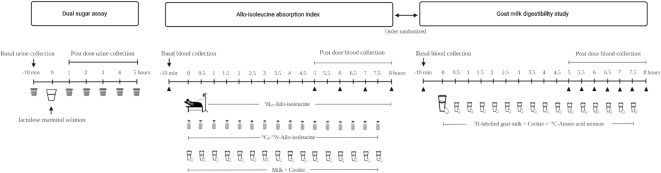
Study experimental protocol followed for dual-sugar assay, allo-isoleucine absorption index, and digestibility of goat milk protein.

After an overnight fast of 10 h, participants reported to the Division of Nutrition of St. John's Research Institute at 08:00 on the study day. A baseline urine sample was collected from each participant, after which an oral solution of lactulose (5 g; Tokyo Chemical Industry) and mannitol (1 g; Merck) in 75 mL of water was administered to them (21). Urine samples were collected hourly after that, up to 5 h postdose (**Figure 1**). Participants were not allowed any food or beverages except water until the end of the experiment. Postdose hourly urine samples were proportionally pooled for 1–2 and 1–5 h, aliquoted, and stored at –20^⁰^C until analysis.

### Allo-isoleucine absorption

A dual differentially labeled stable isotope nonprotein amino acid, allo-isoleucine, study was conducted to assess amino acid absorption (18). On the day of the experiment, participants reported to the metabolic ward at 06:30 after an overnight fast of 10 h. Two indwelling intravenous (IV) cannulas (Jelco 22 G; Medex Medical Ltd.) were secured in each arm, one for IV infusion of the tracer and the other for venous blood sampling. The participants remained in a reclining position with minimal physical activity throughout the experiment. The experimental protocol started at 07:00 and continued for a duration of 8 h. A continuous IV infusion of ^2^H_10_-L-allo-isoleucine (0.3 μM/kg/h, >99%; Cambridge Isotope Laboratories) was started simultaneously with half-hourly oral boluses of ^13^C_6_,^15^N-L-allo-isoleucine (0.3 μM/kg/h, >99%; Cambridge Isotope Laboratories) until the end of the protocol. The participants were provided one-third of their daily sedentary energy and protein requirement (**Table 1**) during the protocol, composed of ultra-heat-treated milk (Goodlife; Karnataka Milk Federation) with beet sugar and protein-free wheat starch cookies (**Supplementary Table 1**). The total meal amount was divided into 16 equal portions, each portion representing a mini meal, which was fed half-hourly from the start of the protocol, to ensure a steady plateau pattern of feeding. A basal blood sample (4 mL) was collected prior to starting the infusion, followed by hourly samples from 5 h onward, until the end of experiment (Figure 1). Whole blood was transferred into EDTA-coated evacuated tubes (Becton Dickenson) and centrifuged at 1098 × *g* at 4^⁰^C for 10 min to separate plasma, which was aliquoted and stored at –80^⁰^C until analysis.

**FIGURE 3:**
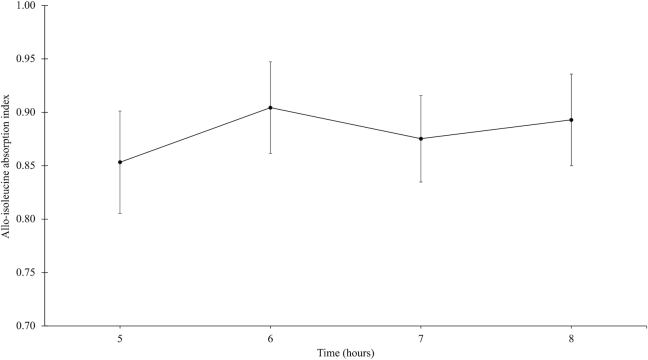
Allo-isoleucine absorption index at plateau (5–8 h) in apparently healthy South Indian women (*n* = 6). Plots represent mean ± SD of allo-isoleucine absorption index.

**TABLE 1:** Nutrient composition of standardized test meal in allo-isoleucine absorption and goat milk digestibility study administered to apparently healthy South Indian women^1^

Meal nutrient^2^	Allo-isoleucine absorption (*n* = 6)	Milk protein digestibility (*n* = 7)
Energy, kcal	672.0 ± 25.1	671.3 ± 30.4
Protein, g	19.7 ± 1.3	26.5 ± 1.7
Carbohydrate, g	80.6 ± 2.3	65.0 ± 2.7
Fat, g	30.4 ± 1.3	34.0 ± 1.8
Protein to energy ratio, %	11.7 ± 0.4	15.8 ± 0.6

1 Values are mean ± SD

2 The nutrient composition presented is representative of the whole test meal, which was divided into smaller portions for half-hourly administration in the plateau feeding protocol.

### Milk protein digestibility

On a separate day, participants reported at 06:30 to the metabolic ward after an overnight fast of 10 h. An indwelling venous cannula (Jelco 22 G; Medex Medical Ltd.) was secured at the beginning of the experiment for blood sample collection. The participants remained in a reclining position with minimal physical activity throughout the experiment. The measurement of true ileal digestibility of the milk protein was performed using the dual-isotope method (15).

The test meal (which was fed in a plateau feeding protocol) consisted of the spray-dried intrinsically labeled milk powder reconstituted in water with beet sugar and protein-free wheat starch cookies (Supplementary Table 1). It provided one-third of the daily sedentary energy requirement (22), and protein was provided at 1.3 g/kg body weight. A higher protein intake in the meal compared with the sedentary protein requirement was given to ensure quantifiable individual plasma IAA enrichments, based on previous measurements of ileal protein digestibility by the same method (19, 23). The nutrient composition of the test meal is provided in Table 1. An 8 h plateau feeding protocol was adopted, in which the test meal was portioned into 21 portions, each portion representing a single mini-meal. Here, an initial priming portion (containing 6 mini-meals) was fed at the beginning of the protocol, followed by single mini-meals every half hour. Meals were warmed in a microwave oven before administration to the participants. One mini-meal aliquot was retained for IAA isotopic enrichment analysis.

The intrinsically ^2^H-labeled milk protein made up the total protein in the test meal, with a trace amount (∼0.1%) contributed by a U-^13^C-labeled algal amino acid mixture (1.25 mg/kg, 98%; Cambridge Isotope Laboratories). The latter served as the standard protein that is fully digested (15, 19) for comparison in the dual-isotope method. ^13^C_11_-L-tryptophan (0.04 mg/kg, 99% purity; Cambridge Isotope Laboratories) was also added to the meal, as it was absent in the labeled algal amino acid mixture. ^13^C_11_-L-tryptophan was added to allow for digestibility measurement at a later date on standardizing an appropriate method.

A basal blood sample (4 mL) was collected prior to the administration of mini-meals, followed by half-hourly samples from 5 h onward, until the end of the experiment (Figure 1). Whole blood was transferred into EDTA-coated anticoagulant evacuated tubes (Becton Dickenson), processed, and stored as described above.

### Analyses of intrinsically labeled fodder and goat milk protein

The ^2^H-IAA enrichment in the fodder, as well as a precipitated aliquot of each day's milk protein and pooled spray-dried milk powder, was measured as described in detail elsewhere (15, 24). Briefly, milk protein was precipitated using trichloroacetic acid (25%, v/v; Sigma-Aldrich), followed by acetone wash (75%, v/v; Merck) and vacuum drying (24). The milk protein precipitate and pooled spray-dried milk powder underwent a gas-phase acid hydrolysis followed by cation exchange purification of the IAAs and derivatization to N-ethoxycarbonyl ethyl esters. The ^2^H-IAA enrichments were measured using GC–pyrolysis–isotope ratio mass spectrometry (Delta V Advantage; Thermo Fisher Scientific) and expressed as parts per million excess (ppme) (15). Baseline ^2^H-IAA abundance was measured in the milk protein collected before the start of the labeled fodder feeding protocol.

### Urine analysis for lactulose and mannitol

Urinary concentrations of lactulose and mannitol were measured as described earlier (25). Briefly, 100 μL of urine sample was deionized with 5 mg of AG 501-X8(D) resin (Bio-Rad) after spiking with ^13^C_6_-mannitol and ^13^C_12_-lactulose as internal standards (50 µL each from of 100 µM/L stock solution; Cambridge Isotope Laboratories) by vortex mixing for an hour and centrifuged at 1098 × *g* at 28^⁰^C. The supernatant was dried, and the sugars were derivatized as their silylated esters and quantified by GC-MS (SQ, 5975; Agilent Technologies) (25). Lactulose (an index of intestinal epithelial barrier/integrity) and mannitol (an index of intestinal absorption) recovery (as percent) pooled samples (2 h and 5 h) were calculated by multiplying the concentration of the sugar with the total volume of urine void and dividing by the dose of the respective sugars. The ratio of lactulose to mannitol recovery provided the lactulose/mannitol ratio (LMR). Eventually, the LMR at 2 h postdose was used since LMR at 2 h and 5 h correlated well (*r*_s _= 0.99, *P *< 0.0001) and 2 h offers a closer representation of small intestinal absorption (21, 26, 27).

### Analyses of plasma and meal IAA enrichment

Isotopic enrichments in plasma and lyophilized test meal samples were measured as explained elsewhere (15). The masses monitored for isotopes of allo-isoleucine accounted for the transamination of the isotopes administered (**Supplementary Table 2**). The allo-isoleucine absorption index was defined as a ratio of isotopic enrichment of oral (^13^C_6_, ^15^N-L-allo-isoleucine) to IV (^2^H_10_-L-allo-isoleucine) tracee in the plasma. In addition to this, the phenylalanine absorption index, as previously estimated (15) using free ^13^C_6_-L-phenylalanine, was computed with free ^13^C_9_-L-phenylalanine, which was part of the free ^13^C IAA mix administered with the present protocol, and calculated as

(1) $$\begin{eqnarray*}
&&\big[ {\rm{Plasm}}{{\rm{a}}^{{\rm{13}}}}{{\rm{C}}_{\rm{9}}}{\rm{ - phenylalanine }}\left( {{\rm{ppme}}} \right)/\nonumber\\
&&\quad {\rm{Mea}}{{\rm{l}}^{{\rm{13}}}}{{\rm{C}}_{\rm{9}}}{\rm{ - phenylalanine}}\,\left( {{\rm{ppme}}} \right) \times {\rm{100}} \big].
\end{eqnarray*}$$

The same equation can be applied to arrive at absorption indices using other free ^13^C IAA plasma to meal ratios.

The true ileal digestibility percentage for each IAA of milk was calculated using the following equation:

(2) $$\begin{eqnarray*}
&&\big[ {{\rm{Plasm}}{{\rm{a}}^{\rm{2}}}{\rm{H - IAA}}\,\left( {{\rm{ppme}}} \right){\rm{/Mea}}{{\rm{l}}^{\rm{2}}}{\rm{H - IAA}}\,\left( {{\rm{ppme}}} \right)} \big]/\nonumber\\
&&\quad\left[ {{\rm{Plasm}}{{\rm{a}}^{{\rm{13}}}}{\rm{C - IAA }}\left( {{\rm{ppme}}} \right){\rm{/Mea}}{{\rm{l}}^{{\rm{13}}}}{\rm{C - IAA }}\left( {{\rm{ppme}}} \right)} \right]\nonumber\\
&&\qquad \times {\rm{100}}.
\end{eqnarray*}$$

### Statistical analysis

Five participants were required to obtain a standard deviation of 1.8 (8) with 95% confidence and 2% precision. All data are reported as arithmetic mean and SD unless specified. Spearman correlation was used to evaluate the associations. For all the comparisons, *P *< 0.05 was considered significant. All calculations were performed with SPSS, version 25 (SPSS, Inc.).

## Results

### Goat milk yield and enrichment

**FIGURE 2:**
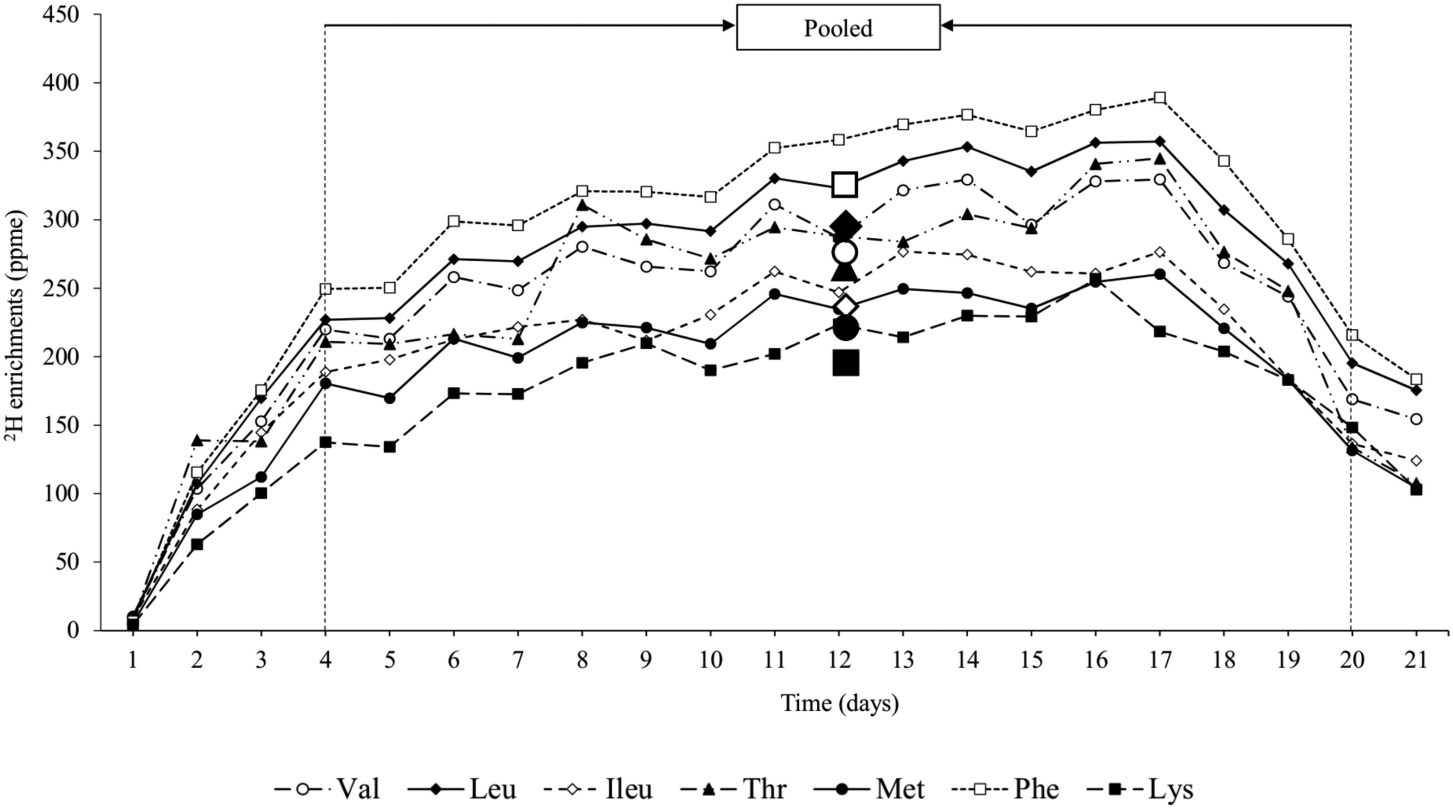
Daily and pooled (4–20 d) ^2^H indispensable amino acid (IAA) enrichments of intrinsically labeled goat milk [parts per million excess (ppme)]. The pooled enrichments are represented using markers corresponding to each IAA. The average IAA ^2^H enrichment of the pooled milk was 257 ppme.

The total yield of intrinsically labeled maize and cowpea fodder was 15 kg and 2.5 kg, respectively. The average ^2^H-IAA enrichment of the fodder was 1607 and 1357 ppme for maize and cowpea, respectively (**Supplementary Figure 2**). The volume of ^2^H-intrinsically labeled milk collected during the study was 14.5 L. The milk collected on the first 3 d and the last day (day 21) was not used for the human digestibility study. The daily and pooled ^2^H-IAA enrichment of milk is represented in **Figure 2**. The average IAA enrichment of pooled goat milk (between 4 and 20 days) was 257 ppme.

### Human intestinal permeability and allo-isoleucine absorption

The participant characteristics for the study are provided in **Table 2**. The mean ± SD hourly lactulose and mannitol excretion for 2-h pooled urine samples was 6.8 ± 2.1 and 216.2 ± 66.5 μM/h, respectively. The mean lactulose and mannitol recovery (percent) and LMR for 2-h pooled samples are provided in **Table 3**, and the LMR ranged from 0.008 to 0.020. The mean ± SD lactulose and mannitol recovery (percent) and LMR for 5-h pooled urine samples were 0.24 ± 0.06%, 16.9 ± 4.4%, and 0.015 ± 0.004, respectively. The allo-isoleucine absorption index at plateau (5–8 h) is provided in **Figure 3**, and the mean allo-isoleucine absorption index is provided in Table 3. The mean interindividual CV of ^13^C_6_,^15^N-L-allo-isoleucine and ^2^H_10_-L- allo-isoleucine enrichment at plateau was 15% and 12%, respectively. The mean ^13^C_9_ phenylalanine absorption index was 24.5 ± 1.6%.

**TABLE 2:** Characteristics of study participants^1^

Variable	Value
Age, y	27.4 ± 4.8
Weight, kg	58.1 ± 3.4
Height, m	1.6 ± 0.02
BMI, kg/m^2^	23.0 ± 1.2

1 Values are mean ± SD, *n* = 7.

**TABLE 3:** Lactulose and mannitol recovery (%), lactulose/mannitol ratio and allo-isoleucine absorption index in apparently healthy South Indian women^1^

Intestinal function parameter^2^	Value
Lactulose recovery, %	0.09 ± 0.03
Mannitol recovery, %	7.90 ± 2.34
Lactulose/mannitol ratio	0.012 ± 0.004
Allo-isoleucine absorption index, %	88.4 ± 3.8

1 Values are mean ± SD.

2 *n* = 7 for lactulose and mannitol recovery, as well as lactulose/mannitol ratio; *n* = 6 for allo-isoleucine absorption index.

### Goat milk protein digestibility

**FIGURE 4:**
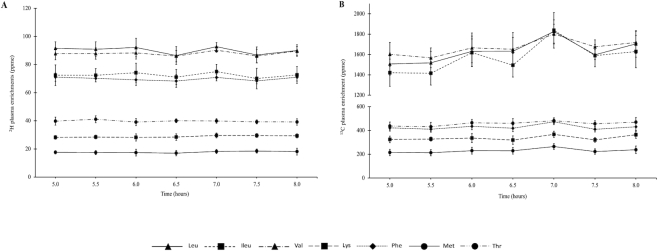
Plasma enrichment of indispensable amino acid (IAA) after consumption of intrinsically labeled spray-dried goat milk test meal in apparently healthy South Indian women. (A) Plasma appearance of ^2^H. (B) ^13^C isotopic enrichments of IAA [parts per million excess (ppme)] at plateau (*n* = 7). Plots represent mean ± SE of ^2^H and ^13^C plasma IAA enrichments.

The mean ^2^H and ^13^C IAA enrichments (ppme) of the protein in the test meals are provided in **Supplementary Table 3**. The ^2^H and ^13^C enrichments of each plasma IAA at plateau (5–8 h) are represented in **Figure 4**. The mean interindividual CV of ^2^H and ^13^C plasma enrichment at plateau was 15% and 19%, respectively. The CVs for both ^2^H and ^13^C plasma enrichments were lowest for phenylalanine at 10% and 14%, respectively, and highest for methionine at 23% and 25%, respectively. The mean ± SD true ileal digestibility of spray-dried milk powder was 94.0 ± 2.9% and ranged from 89.9% for threonine to 97.9% for methionine (**Table 4**). There was a significant inverse correlation (*r*_s_ = –0.93, *P* = 0.008) between the LMR and allo-isoleucine absorption index (*r*^2 ^= 0.49, slope = –6.9323 and intercept = +0.9714) but not between either of these variables and phenylalanine absorption index or IAA digestibility.

**TABLE 4:** True ileal digestibility of spray-dried goat milk powder in apparently healthy South Indian women^1^

Amino acid	True ileal digestibility, % (*n* = 7)
Methionine	97.9 ± 1.8
Phenylalanine	93.0 ± 2.4
Threonine	89.9 ± 1.2
Lysine	93.0 ± 2.1
Leucine	95.2 ± 2.5
Isoleucine	97.3 ± 1.4
Valine	92.0 ± 2.7
Mean	94.0 ± 2.9

1 Values are mean ± SD.

## Discussion

This study determined the true ileal IAA digestibility of intrinsically labeled, spray-dried goat milk powder, using the dual stable isotope tracer technique, in healthy South Indian WRA whose intestinal integrity was assessed by a dual-sugar test and amino acid absorption estimated using a nonprotein amino acid (allo-isoleucine). The estimated mean true ileal IAA digestibility of goat milk protein was 94.0% in these women, with normal intestinal function as assessed by LMR and allo-isoleucine absorption index.

### Goat milk protein digestibility

The mean true ileal IAA digestibility of goat milk protein (94.0%) was similar to that of cow milk protein isolate and skimmed milk protein (95.1% and 95.5%) in healthy French adults (8, 28). It also compared reasonably well (94.0% compared with 97.2%) with the true ileal digestibility of goat milk protein concentrate in rats (29). The individual IAA digestibility was within the range (93–100%) of previously reported values (8). The mean true ileal IAA digestibility of goat milk protein was similar to that of other ASF and greater than plant source protein determined in healthy Indian adults of both sexes by the dual-isotope tracer technique (15, 19, 30). The high digestibility of milk protein makes it an important ASF in populations that are predominantly vegetarian and in countries like India, where frequency of meat consumption is low, to alleviate the risk of quality protein inadequacy (6, 31).

The intrinsic labeling of dietary protein with stable isotopes allows for the precise measurement of true ileal protein digestibility. Previously, goat or cow milk has been labeled with ^2^H and ^15^N by oral administration of ^2^H_2_O or (^15^NH_4_)_2_SO_4_ to the lactating animal, for use in direct orofecal or true ileal protein/IAA digestibility measurements, through killing (rats) or nasoileal intubation (humans), respectively (8, 28, 32). However, milk obtained in this manner is mostly likely to be labeled on either the α-H or α-N moiety of the constituent IAAs (33, 34) through transamination, and these are prone to losses in the body by the same process. The isotopic label lost through transamination cannot be traced to its parent molecule, thereby limiting its use for the dual-isotope tracer approach. Hence, for this study, it was necessary to label the fodder fed to the goat with ^2^H_2_O, which leads to the stable incorporation of ^2^H at random positions on the amino acid carbon backbone efficiently labeling all amino acids, with minimal losses of α-^2^H that could be corrected using a transamination correction factor (TCF) when labeled milk is administered in humans (15). However, the TCF was not applied for the human digestibility calculation in this study, since transamination losses of α-^2^H have already occurred in the lactating goat (35).

### Intestinal permeability by the dual-sugar assay

The percentage recovery of lactulose and mannitol and the LMR at 2 h was 0.09%, 7.9%, and 0.012, respectively, and the mean allo-isoleucine absorption index was 88%. From the LMR values, it is reasonable to consider that in the present study, women had normal intestinal integrity, because this was lower than the ratio obtained (0.012 compared with 0.020) in a previous study on US adults of either sex, employing the same dose and protocol (21). This could be attributed to a lower lactulose recovery (0.09% compared with 0.20%) in the present study. The LMR obtained at 5 h was lower in the present study compared with earlier studies in Indian women and those residing in the United Kingdom (0.014 compared with 0.114 and 0.038), owing to a higher lactulose excretion (0.24% compared with 1.8% and 0.38%) compared with the present study (13, 14). The possibility of subclinical EE with intestinal epithelial damage leading to secondary impairment of digestion or absorption was therefore unlikely in women in the present study.

### Amino acid absorption: Allo-isoleucine and phenylalanine absorption index

The mean allo-isoleucine absorption index obtained was similar (88% compared with 80%) to the estimated value in healthy US adults (18). Absorption of individual test and reference amino acids is assumed to be equivalent (after protein digestion) in the dual-tracer approach for protein digestibility. Because this equivalent absorption cancels out, the dual-isotope method gives a readout of true ileal digestibility but not absorption. The absorption is also assumed to be maximum; if a value lower than this is used, the net transfer of amino acids into the body could be lower. This is important, as earlier reports have shown mean glycine (amino acid) and glycylglycine (peptide) absorption to be lower by 31% in healthy asymptomatic Indian men compared with age-matched English men (36). Here, the absorption of a nonprotein amino acid (allo-isoleucine), which is assumed to be transported by the same secondary active transporters (Na^+^ dependent, B^0^AT1) as neutral amino acids (7 of the 9 essential amino acids) across the intestinal epithelium, was close to 100% (37), which is the normal absorptive capacity of the proximal small intestine epithelial amino acid transporters in the participants. Furthermore, the small bowel reserve capacity for absorption is unlikely to be affected unless extensive gut damage occurs or surgical resections are performed (38, 39). Therefore, even if absorptive losses are observed, their clinical relevance needs to be established.

Traditionally, amino acid absorption has been studied directly using intestinal infusion studies (40) that are invasive or by the “glycine tolerance test” (41), which has multiple metabolic fates precluding accurate measurement. Stable amino acid isotopes can be exploited to estimate an absorption index. For example, the use of the plasma to meal ratio of enrichments of orally administered ^13^C_6_ phenylalanine and ^13^C_9_ phenylalanine in the present study (15) is an approximation for intestinal absorption of this essential amino acid but includes first-pass metabolism by splanchnic tissues, which is assumed to be less variable in a fed steady state (42). The method used in the present study is a better reflection of intestinal absorption, as amino acid isomers such as D-phenylalanine and L-allo-isoleucine are thought to be metabolically inert, with no metabolic fate except for oxidation in humans (18, 43). Although L-allo-isoleucine, a by-product of L-isoleucine transamination, could reconvert to L-isoleucine, this might not be quantitatively significant considering that it is a poor substrate for branched-chain amino transferases (44, 45). Nevertheless, the robustness and sensitivity of these methods to assess impairment in intestinal amino acid absorption need to be tested in clinical populations with a variable degree of amino acid malabsorption.

### Associations between intestinal permeability, amino acid absorption, and protein digestibility

The LMR in the present study showed a significant inverse relation with the allo-isoleucine absorption index. This finding implies that disrupted intestinal barrier function may impair amino acid absorption, which needs further evaluation involving participants with a wider distribution of LMR or abnormal values. LMR and allo-isoleucine absorption did not correlate with either the mean digestibility or individual IAA digestibility. Experimental evidence indicates that irradiated minipigs with intestinal mucosal barrier disruption (similar to EE) impairing absorption and citrulline production did not show a significant decrease in protein digestion, thereby pointing to the reserve capacity of the pancreas whose cumulative postprandial enzyme production (10- to 15-fold) far exceeds the quantity required for digestion under normal physiologic conditions (17, 46). Otherwise, the digestibility of milk protein is mainly dependent on the quantity of antinutritional components produced during processing (9) or the antinutrients contributed by other ingredients in a mixed meal.

### Strengths and limitations

The strength of this study lies in the near-simultaneous estimation of intestinal function, especially amino acid absorption, along with the determination of protein digestibility, in an LMIC group of participants. A limitation is that the absorption of allo-isoleucine is assumed to represent neutral amino acid transporters, particularly the absorption of branched-chain amino acids, which is reasonable, as it is an isomer of isoleucine (37); however, the function of peptide transporters that demonstrate a kinetic advantage over amino acid transporters was not tested (47). Measurement of the allo-isoleucine absorption index is invasive and therefore has limited application in vulnerable populations, particularly children. The dual-isotope tracer technique is an indirect method and still needs direct validation against amino acid balances measured by nasoileal intubation or ileostomates, but the present study values compared well with those measured by nasoileal intubation (8, 28). In addition, the assessment of other biomarkers of EE could complement the intestinal function measurements in this study.

### Conclusion

In conclusion, this study provides true ileal IAA digestibility of spray-dried goat milk powder in healthy South Indian women with normal intestinal function, which is comparable to estimates from HICs and adds to the expanding literature for protein quality assessment using the Digestible Indispensable Amino Acid Score (DIAAS).

## Supplementary Material

nqaa400_Supplemental_Files

## Data Availability

Data described in the manuscript, code book, and analytic code will be made available upon request to the corresponding author.

## Notes

This research work was supported by The Wellcome Trust/DBT India Alliance through the Margdarshi Fellowship (grant IA/M/14/1/M/501681) awarded to AVK.

This study was registered at Clinical Trials Registry of India (CTRI) as CTRI/2020/04/024512.

Supplemental Tables 1–3 and Supplemental Figures 1 and 2 are available from the “Supplementary data” link in the online posting of the article and from the same link in the online table of contents at https://academic.oup.com/ajcn/.

SK and NS contributed equally to this article and share first authorship.

Abbreviations used: ASF, animal source food; EE, environmental enteropathy; ELU, Experimental Livestock Unit; HIC, high-income country; IAA, indispensable amino acid; IV, intravenous; LMIC, low- and middle-income country; LMR, lactulose/mannitol ratio; NIANP, National Institute of Animal Nutrition and Physiology; ppme, parts per million excess; *r*_s_, Spearman's Rho; TCF, transamination correction factor; WRA, women of reproductive age.
