# Supplementary material for: Goat milk protein digestibility in relation to intestinal function
Source: Am J Clin Nutr. 2021 Mar 1;113(4):845–53. doi: 10.1093/ajcn/nqaa400 (PMC8023838; doi:10.1093/ajcn/nqaa400)
Supplement: nqaa400_Supplemental_Files [file nqaa400_supplemental_files.zip › Supplemental file for reviewers only.pdf]

US 20170097335A1

(19) **United States**

(12) **Patent Application Publication**

(10) **Pub. No.: US 2017/0097335 A1**

(43) **Pub. Date: Apr. 6, 2017**

Deutz et al.

(54) **METHODS FOR DIAGNOSING IMPAIRED ABSORPTION OF AMINO ACIDS, MONOSACCHARIDES AND FATTY ACIDS**

(71) Applicants: **Nicolaas E. Deutz**, College Station, TX (US); **Gerdien C. Ligthart-Melis**, Almere (NL); **Marielle Engelen**, College Station, TX (US); **Gabriella Ten Have**, Montgomery, TX (US)

(72) Inventors: **Nicolaas E. Deutz**, College Station, TX (US); **Gerdien C. Ligthart-Melis**, Almere (NL); **Marielle Engelen**, College Station, TX (US); **Gabriella Ten Have**, Montgomery, TX (US)

(73) Assignee: **The Texas A&M University System**, College Station, TX (US)

(21) Appl. No.: **15/287,420**

(22) Filed: **Oct. 6, 2016**

Related U.S. Application Data

(60) Provisional application No. 62/237,794, filed on Oct. 6, 2015.

Publication Classification

(51) **Int. Cl.**

G01N 33/50 (2006.01)

G06F 19/00 (2006.01)

G01N 33/58 (2006.01)

(52) **U.S. Cl.**

CPC ***G01N 33/5091*** (2013.01); ***G01N 33/58*** (2013.01); ***G06F 19/366*** (2013.01); *G01N 2458/15* (2013.01); *G01N 2800/02* (2013.01)

(57) **ABSTRACT**

Provided herein are methods for diagnosing impaired nutrient absorption or determining a nutrient absorption level in an individual. A first stable absorption isotopologue of a nutrient and a second stable absorption isotopologue of the same nutrient are administered orally and intravenously, respectively, to the individual. Amounts of the first and second isotopologues are both measured in blood samples drawn periodically and a ratio of the first isotopologue to the second isotopologue is calculated providing a level of nutrient absorption. The results are compared with a healthy control or an ideal value of 1.0. The ratio less than that of a healthy control or significantly lower than 1.0 indicates an impaired nutrient absorption in the individual.

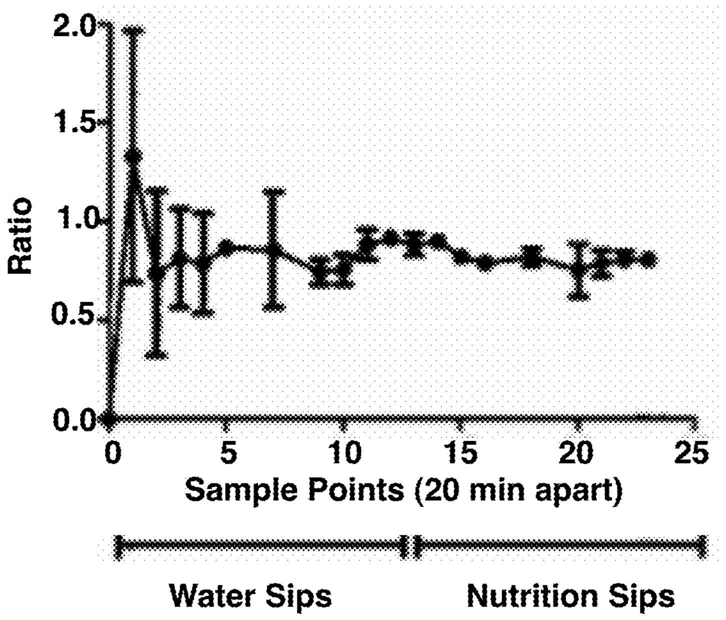
2.0
1.5
1.0
0.5
0.0
Ratio
0
5
10
15
20
25
Sample Points (20 min apart)
Water Sips
Nutrition Sips

FIG. 1

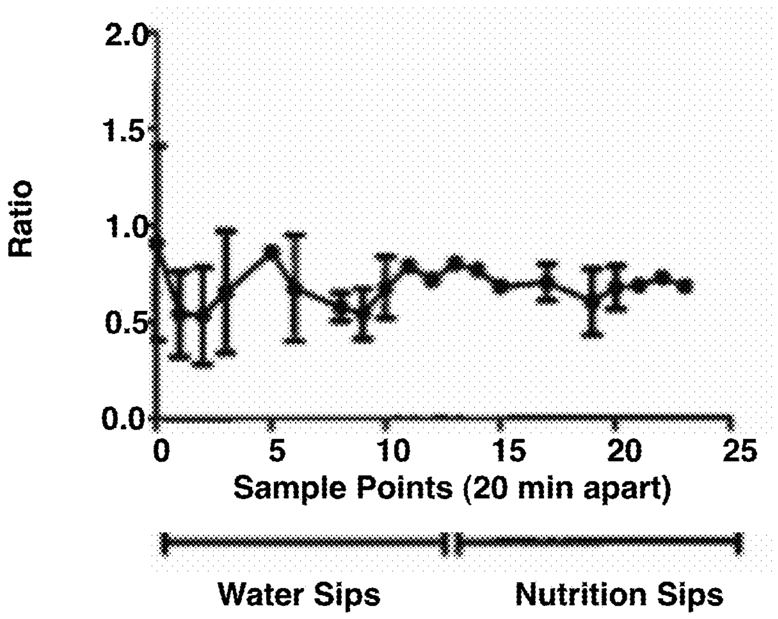
2.0
1.5
1.0
0.5
0.0
Ratio
0
5
10
15
20
25
Sample Points (20 min apart)
Water Sips
Nutrition Sips

FIG. 2

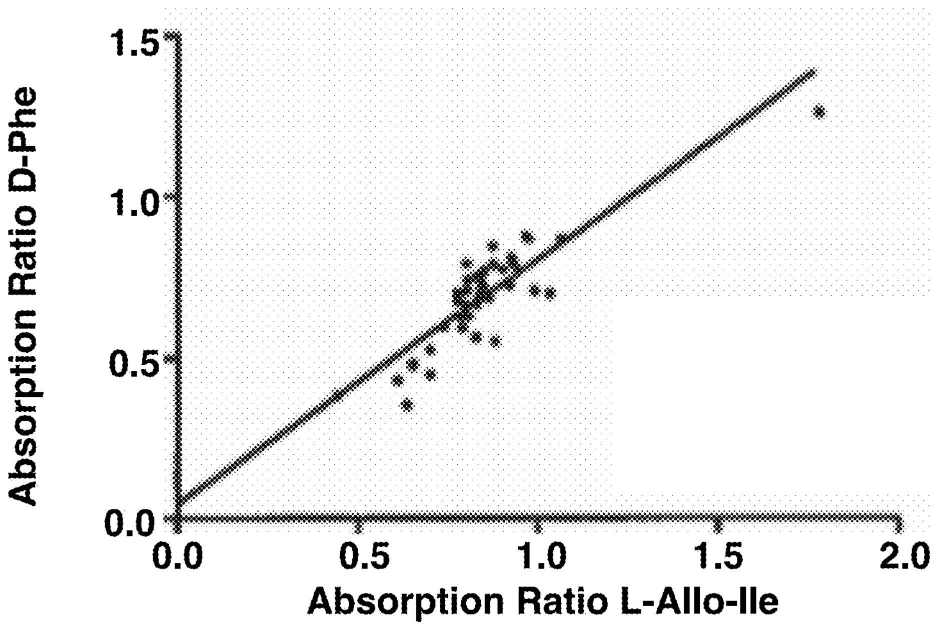
1.5
1.0
0.5
0.0
Absorption Ratio D-Phe
0.0
0.5
1.0
1.5
2.0
Absorption Ratio L-Allo-Ile

FIG. 3

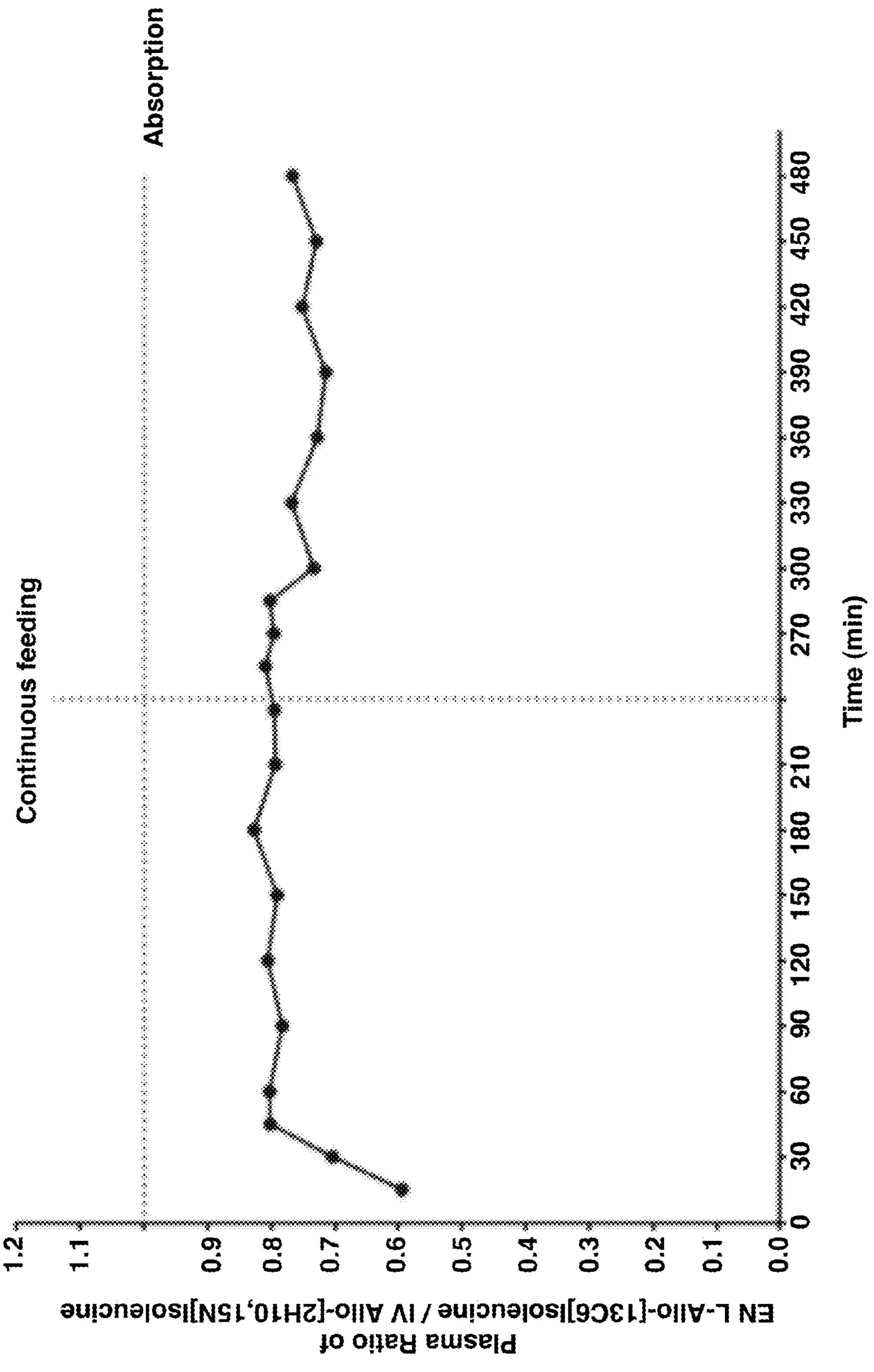
Plasma Ratio of
EN L-Allo-[13C6]isoleucine / IV Allo-[2H10,15N]isoleucine
1.2
1.1
0.9
0.8
0.7
0.6
0.5
0.4
0.3
0.2
0.1
0.0
Continuous feeding
Absorption
0
30
60
90
120
150
180
210
270
300
330
360
390
420
450
480
Time (min)

FIG. 4

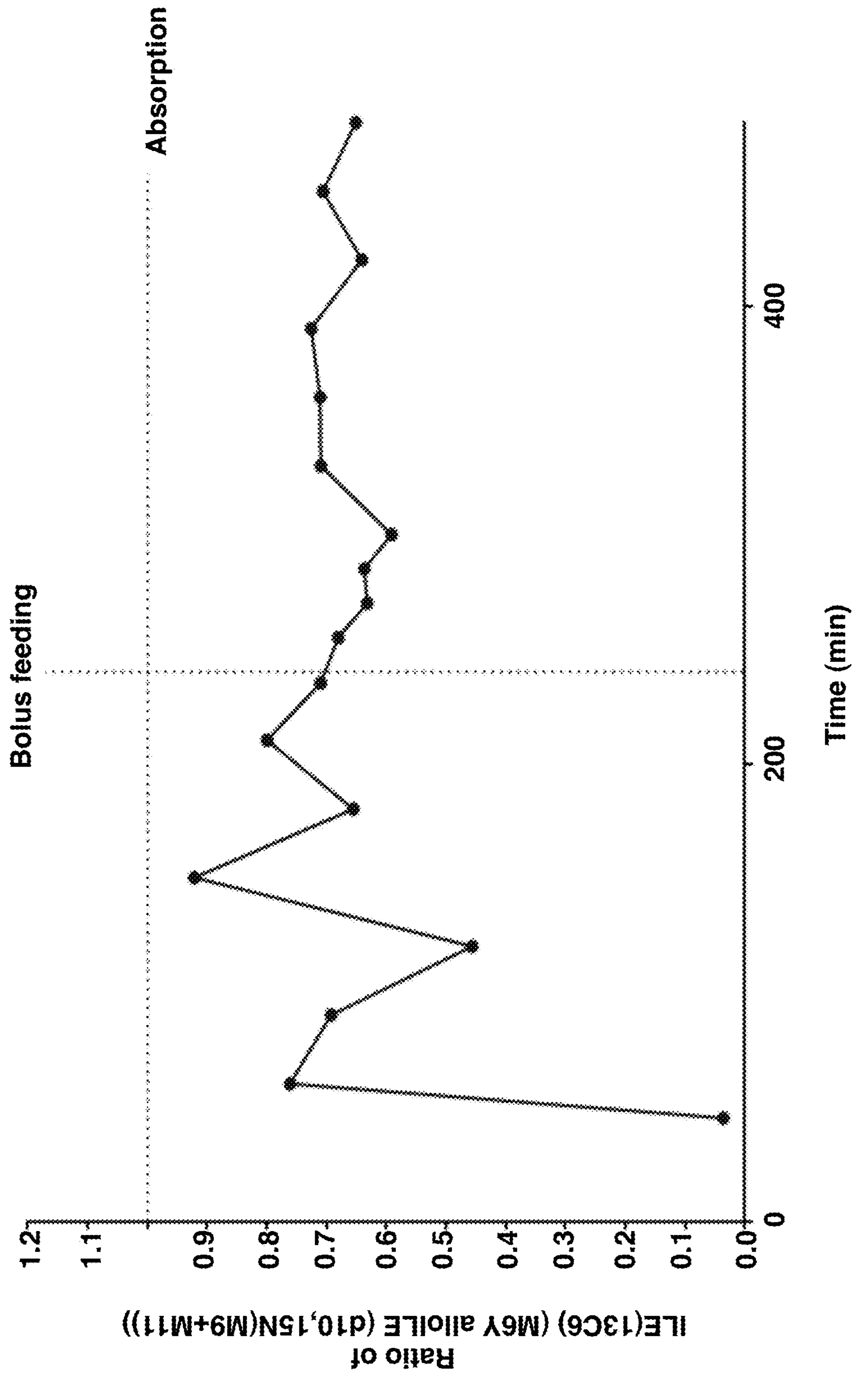
Bolus feeding
Absorption
Ratio of
ILE(13C6) (M6Y alloILE (d10,15N(M9+M11))
1.2
1.1
0.9
0.8
0.7
0.6
0.5
0.4
0.3
0.2
0.1
0.0
0
200
400
Time (min)

FIG. 5

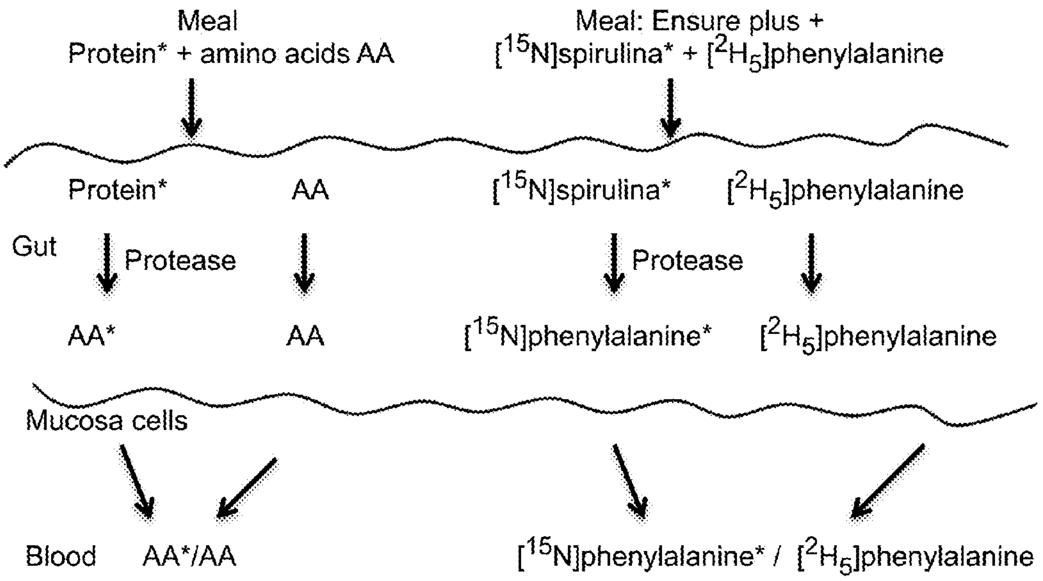
Meal
Protein* + amino acids AA
Meal: Ensure plus +
[^{15}N]spirulina* + [^{2}H$_{5}$]phenylalanine
Protein*
AA
[^{15}N]spirulina*
[^{2}H$_{5}$]phenylalanine
Gut
Protease
Protease
AA*
AA
[^{15}N]phenylalanine*
[^{2}H$_{5}$]phenylalanine
Mucosa cells
Blood
AA*/AA
[^{15}N]phenylalanine* / [^{2}H$_{5}$]phenylalanine

FIG. 6

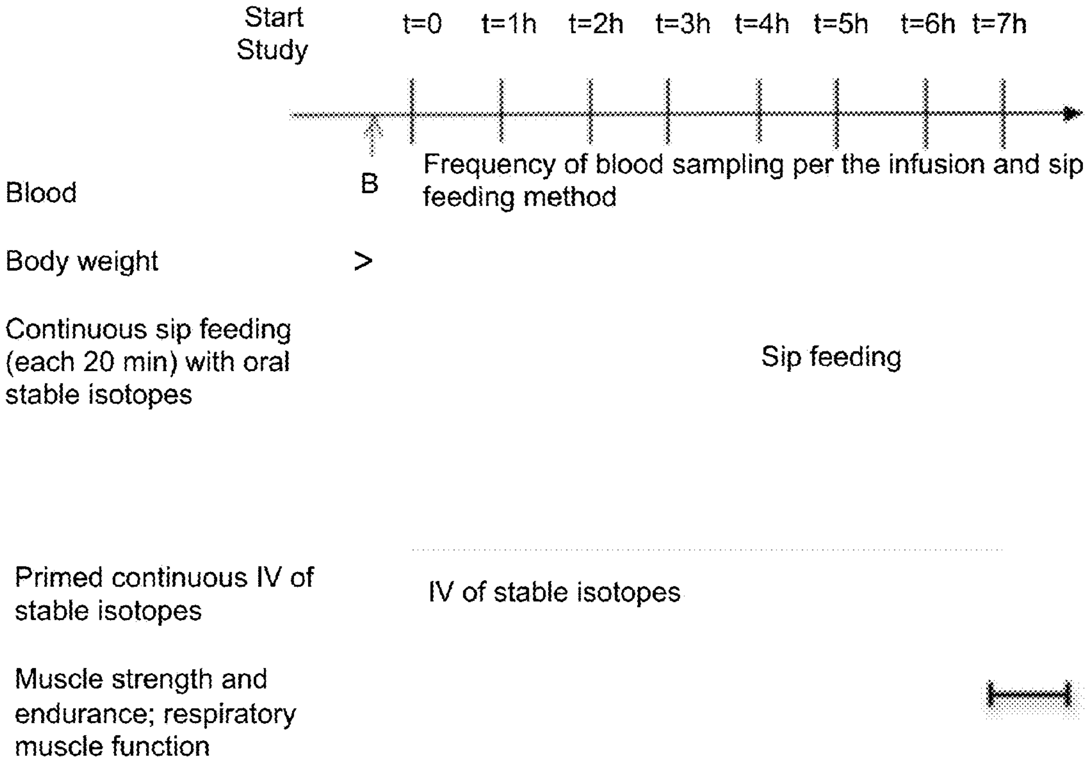
Start Study
t=0
t=1h
t=2h
t=3h
t=4h
t=5h
t=6h
t=7h
Blood
B
Frequency of blood sampling per the infusion and sip feeding method
Body weight
>
Continuous sip feeding (each 20 min) with oral stable isotopes
Sip feeding
Primed continuous IV of stable isotopes
IV of stable isotopes
Muscle strength and endurance; respiratory muscle function

FIG. 7

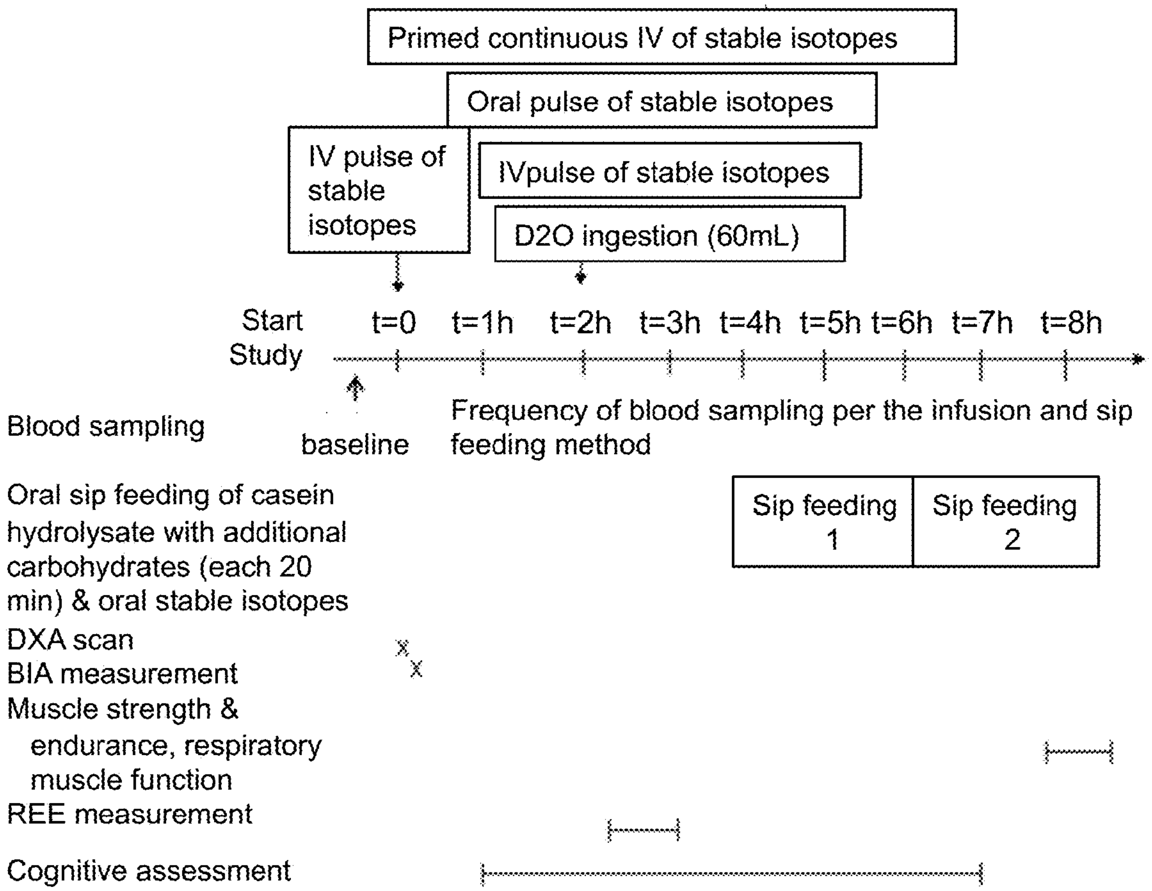
Primed continuous IV of stable isotopes
Oral pulse of stable isotopes
IV pulse of stable isotopes
IVpulse of stable isotopes
D2O ingestion (60mL)
Start Study
t=0
t=1h
t=2h
t=3h
t=4h
t=5h
t=6h
t=7h
t=8h
Blood sampling
baseline
Frequency of blood sampling per the infusion and sip feeding method
Oral sip feeding of casein hydrolysate with additional carbohydrates (each 20 min) & oral stable isotopes
Sip feeding 1
Sip feeding 2
DXA scan
BIA measurement
x
x
Muscle strength & endurance, respiratory muscle function
REE measurement
Cognitive assessment

FIG. 8

METHODS FOR DIAGNOSING IMPAIRED ABSORPTION OF AMINO ACIDS, MONOSACCHARIDES AND FATTY ACIDS

CROSS-REFERENCE TO RELATED APPLICATIONS

[0001] This non-provisional application claims benefit of priority under 37 U.S.C. §119(e) of provisional application U.S. Ser. No. 62/237,794, filed Oct. 6, 2015, the entirety of which is hereby incorporated by reference.

BACKGROUND OF THE INVENTION

[0002] Field of the invention

[0003] The present invention generally relates to the field of diagnostic tools to assess the metabolism of nutrients. More specifically, the present invention relates to a method for diagnosing impaired absorption of amino acids, monosaccharides and fatty acids using stable isotopologues of amino acids.

[0004] Description of the Related Art

[0005] Intestinal absorption of amino acids, monosaccharides and fatty acids are essential for human metabolism. Impaired absorption of amino acids via the intestinal wall, which occurs in patients with intestinal failure, can lead to involuntary, potentially lethal weight loss and muscle wasting. Therefore, it is imperative to diagnose impaired absorption of these nutrients before it causes significant damage to a patient's health.

[0006] Current methods, however, only measure combined digestion and absorption of nutrients and thus cannot distinguish impaired absorption from impaired digestion. While impaired digestion may affect a patient's health, it can be easily treated with supplementation of digestive enzymes. Impaired absorption, on the other hand, may require more medical attention. In addition, these methods often show accuracies in determining the exact value of nutrients absorption.

[0007] For instance, a commonly used method to determine protein digestion/absorption includes feeding the patients intrinsically labeled proteins. In this method, plants or animals are fed with labeled amino acids. The patient then consumes resultant proteins from plants, meat or milk. The blood of the patient can then be sampled to measure the uptake of protein by the intestine. However, besides not being able to distinguish impaired absorption from impaired digestion, it is challenging to obtain large amount of proteins that contain sufficiently labeled amino acids for accurate detection. Furthermore, fecal loss analysis, which is considered as the gold standard for analysis of nutrient metabolism, is a poor indicator of the uptake of amino acids since unabsorbed amino acids can be partly metabolized by bacteria into ammonia and subsequently absorbed by the colon mucosa, resulting in undetectable nitrogen loss and false results for amino acid metabolism.

[0008] Thus, there is a recognized need in the art for a method of accurately and distinguishably measuring nutrient, such as, amino acids, monosaccharides and fatty acids absorption. Particularly, the prior art is deficient in these aspects. The present invention fulfills this long standing need and desire in the art.

SUMMARY OF THE INVENTION

[0009] The present invention is directed to a method for diagnosing impaired nutrient absorption in an individual. In the method a first stable absorption isotopologue of the nutrient is administered orally to the individual and a second stable absorption isotopologue of the same nutrient is administered intravenously to the individual. Blood samples are drawn periodically from the individual and an amount of each isotopologue is measured in each sample. A ratio of the first isotopologue to the second isotopologue is calculated in each sample. The ratio calculated for the individual is compared to a ratio calculated for a healthy control; where, when the ratio calculated for the individual is less than the ratio for the healthy control, impaired absorption of the nutrient in said individual is present. The present invention is directed to a related method comprising further steps. In the related method the nutrient production, consumption and metabolism are analyzed and a metabolic phenotype based on the analysis is produced.

[0010] The present invention also is directed to a method for determining an intestinal absorption level of a nutrient in an individual. In the method a first stable absorption isotopologue of the nutrient is administered enterally to the individual and a second stable absorption isotopologue of the same nutrient is administered intravenously to the individual. Blood samples are drawn periodically from the individual and an amount of the first isotopologue and of the second isotopologue is measured in each sample. A ratio of the first isotopologue to the second isotopologue is calculated in each sample. The present invention is directed to a related method comprising further steps. In the related method a ratio of the first isotopologue to the second isotopologue is calculated after administration to a healthy control. The ratio calculated for the individual is compared to the ratio calculated for the healthy control; where a decrease in the ratio for the individual compared to the ratio for the healthy control comprises a diagnosis of impaired absorption of the nutrient in the individual.

[0011] Other and further aspects, features, benefits, and advantages of the present invention will be apparent from the following description of the presently preferred embodiments of the invention given for the purpose of disclosure.

BRIEF DESCRIPTION OF THE DRAWINGS

[0012] So that the matter in which the above-recited features, advantages and objects of the invention, as well as others that will become clear, are attained and can be understood in detail, more particular descriptions of the invention briefly summarized above may be by reference to certain embodiments thereof that are illustrated in the appended drawings. These drawings form a part of the specification. It is to be noted, however, that the appended drawings illustrate preferred embodiments of the invention and therefore are not to be considered limiting in their scope.

[0013] FIG. **1** shows the absorption ratios between orally and intravenously provided isotopologue(s) of L-allo-isoleucine in each sample for measurement of amino acid absorption (mean±SD).

[0014] FIG. **2** shows the absorption ratios between orally and intravenously provided isotopologue(s) of D-Phenylalanine in each sample for measurement of protein absorption (mean±SD.

[0015] FIG. **3** shows the statistical correlation absorption ratios of L-allo-isoleucine versus D-Phenylalanine. Statistics: Pearson r is 0.9076, 95% confidence interval is 0.8359 to 0.9488 and P value (two-tailed) is <0.0001.

[0016] FIG. **4** shows the ratios between enterally and intravenously provided isotopologues of L-allo-isoleucine in the blood samples from a pig with a continuous enteral feeding for 4 hours.

[0017] FIG. **5** shows the ratios between enterally and intravenously provided isotopologues of L-allo-isoleucine in the blood samples from a pig after a bolus feeding.

[0018] FIG. **6** illustrates the principle to measure protein digestibility using ^{15}N-labeled spirulina protein. * identifies the path of a generic protein or of the ^{15}N-labeled spirulina protein.

[0019] FIG. **7** illustrates the protocol for determining protein digestion and absorption in chronic heart failure (CHF) and chronic obstructive pulmonary disease (COPD) patients.

[0020] FIG. **8** illustrates the protocol for determining the anabolic effect of high-quality protein sip feeding in COPD, CHF and cancer patients

DETAILED DESCRIPTION OF THE INVENTION

[0021] As used herein in the specification, "a" or "an" may mean one or more. As used herein in the claim(s), when used in conjunction with the word "comprising", the words "a" or "an" may mean one or more than one.

[0022] As used herein "another" or "other" may mean at least a second or more of the same or different claim element or components thereof. Similarly, the word "or" is intended to include "and" unless the context clearly indicates otherwise. "Comprise" means "include."

[0023] As used herein, the term "about" refers to a numeric value, including, for example, whole numbers, fractions, and percentages, whether or not explicitly indicated. The term "about" generally refers to a range of numerical values (e.g., +/−5-10% of the recited value) that one of ordinary skill in the art would consider equivalent to the recited value (e.g., having the same function or result). In some instances, the term "about" may include numerical values that are rounded to the nearest significant figure.

[0024] As used herein, the terms "individual", "patient", "subject" or "healthy control", refer to any human or non-human mammal for which a nutrient absorption level or ratio is determined, calculated or measured.

[0025] In one embodiment of the present invention, there is provided a method for diagnosing impaired absorption of a nutrient in an individual, comprising the steps of administering a first stable absorption isotopologue of said nutrient orally to the individual; administering a second stable absorption isotopologue of the same nutrient intravenously to the individual; drawing blood samples periodically from said individual; measuring an amount of the first isotopologue and of the second isotopologue in each sample; calculating a ratio of the orally administered isotopologue to the intravenously administered isotopologue in each sample; and comparing the ratio calculated for the individual to a ratio calculated for a healthy control; wherein, when the ratio calculated for the individual is less than the ratio for the healthy control, impaired absorption of the nutrient in the individual is present.

[0026] Further to this embodiment the method comprises analyzing the nutrient production, consumption and metabolism; and producing a metabolic phenotype based on the analysis. In this further embodiment the individual may have chronic heart failure, chronic obstructive pulmonary disease, a cancer, a cognitive dysfunction, or may be a bariatric pre-operative or post-operative patient. In both embodiments the calculating step may comprise applying a non-compartmental model to the measured amounts of the first isotopologue and the second isotopologue. Also in both embodiments the ratio of impaired absorption may be 1.0 or less in the individual. In addition the first labeled nutrient and the second labeled nutrient may be administered simultaneously or the second labeled nutrient may be administered within 8 hours of the first labeled nutrient.

[0027] In both embodiments an isotope comprising the isotopologue may be ^{2}H, ^{13}C, or ^{15}N. Representative examples of a nutrient which can be examined using this method include, but are not limited to, an amino acid, carbohydrate, fat, or a combination thereof that is not metabolized in the gut of the individual. Also, non-limiting examples of an isotope comprising the isotopologue may be ^{2}H, ^{13}C, or ^{15}N.

[0028] In a preferred aspect of this embodiment, the first and second isotopologues of the amino acid may comprise L-alto-[$^{2}H_{10}$, ^{15}M-isoleucine, L-alto-[$^{13}C_6$]-isoleucine, D-1-[^{13}C]phenylalanine, D-[$^{13}C_6$]phenylalanine, D-[$^{2}H_5$]-phenylalanine, D-[2-^{2}H]phenylalanine or a combination thereof. In another aspect representative examples of the isotopologues of the carbohydrate may comprise an isotope of 3-O-methylglucose or an isotope of non-metabolized analogues thereof or a combination thereof. In yet another aspect representative examples of the isotopologues of fat may comprise an isotope of heptadecanoic acid an isotope of non-metabolized analogues thereof or a combination thereof.

[0029] In another embodiment of the present invention, there is provided a method for determining an intestinal absorption level of a nutrient in an individual, comprising the steps of administering a first stable absorption isotopologue of the nutrient enterally to the individual; administering a second stable absorption isotopologue of the same nutrient intravenously to the patient; drawing blood samples periodically from the patient; measuring an amount of the first isotopologue and of the second isotopologue in each sample; and calculating a ratio of the first isotopologue to the second isotopologue in each sample, the ratio correlating to the intestinal absorption level in the individual.

[0030] Further to this embodiment the method comprises calculating a ratio of the first isotopologue to the second isotopologue after administration to a healthy control; and comparing the ratio calculated for the individual to the ratio calculated for the healthy control; where a decrease in the ratio calculated for the individual compared to the ratio for the healthy control comprises a diagnosis of impaired absorption of the nutrient in the individual. In this further embodiment the ratio in the individual may be 1.0 or D-[$^{13}C_6$]-phenylalanine, D-[$^{2}H_5$]-phenylalanine, D-[2-^{2}H] phenylalanine or a less. In both embodiments the calculating step may comprise applying a non-compartmental model to the measured amounts of the first isotopologue and the second isotopologue.

[0031] In both embodiments an isotope comprising the isotopologue may be ^{2}H, ^{13}C, or ^{15}N. Preferably in both

embodiments an isotopologue of the amino acid may comprise L-allo-[$^{2}H_{10}$, ^{15}M-isoleucine, L-allo-[$^{13}C_{6}$]-isoleucine, D-1-[^{13}C]phenylalanine, combination thereof. Alternatively, representative examples of the isotopologue of a carbohydrate or of a fat may be those as described supra.

[0032] Provided herein is a method for diagnosing impaired absorption of amino acids, monosaccharides, and fatty acids nutrients in an individual, patient or subject. Preferably, the method utilizes nutrients substrates that are not metabolized in the gut or liver. Impaired absorption of these nutrients is associated with multiple disease states, including, but not limited to intestinal failure, mucosa cell membrane pathologies or a combination thereof. Impaired absorption is determined by calculating a ratio of the amount of a first, stable labeled nutrient measured in one or more blood samples subsequently drawn after enteral, for example, oral, administration to the amount of a second, stable differently labeled same nutrient after intravenous administration measured in the blood sample(s). Calculated ratios of about 1 or less, as compared to a healthy individual (control), are indicative of or correlate to impaired absorption in the subject. A blood sample drawn from the individual, subject, patient, or control prior to administration of the labeled nutrients provides a baseline for natural enrichment of plasma water and metabolites. The absorption ratio for the healthy individual is obtained in the same manner as for the subject or patient.

[0033] The administered nutrients comprise isotopologue combinations labeled with, for example, ^{2}H, ^{13}C, ^{15}N isotopes. The isotopologue-labeled nutrients may be administered simultaneously or the intravenous administration may occur within 8 hours of oral administration. The amount of label on the nutrient and the doses of labeled nutrients administered are well within the purview of one of ordinary skill in the art.

[0034] The methods provided herein are useful to monitor the muscle wasting that that occur in pathophysiological conditions, such as, but not limited to, chronic heart failure, chronic obstructive pulmonary disease and cancers. Monitoring protein digestion and absorption of different meals, for example, enables one of ordinary skill in the art to determine the actual protein requirements in individual patient. Thus, on an individual patient basis, a diet or nutritional supplementation plan effective to stop ongoing muscle loss and even to restore muscle mass from progressive muscle wasting can be designed. Moreover, monitoring the digestibility of protein meals enriched with nutrients effective to stimulate the metabolism of, for example, serotonin, is useful in studying neuropsychological functions and metabolic pathways during aging. Furthermore, using the described methods and genomics, transcriptomics, proteomics, metabolomic, and fuxomics technologies to monitorhow foods and nutrients are digested, absorbed and metabolized when weight is stable and during weight loss induced by bariatric surgery are useful to identify what causes individual differences in weight loss. Thus, post-surgical weight loss plans can be optimized for each patient.

[0035] These methods also are useful to examine various responses to a defined meal. For example, digestion and absorption may be quantified to monitor loss of nutrients. In another example, the anabolic threshold when protein breakdown equals protein synthesis in conditions where the response and the relation between protein intake and net protein synthesis are critical may be determined.

[0036] The following examples are included to demonstrate preferred embodiments of the invention. It should be appreciated by those of skill in the art that the techniques disclosed in the examples which follow represent techniques discovered by the inventor to function well in the practice of the invention, and thus can be considered to constitute preferred modes for its practice. However, those of skill in the art should, in light of the present disclosure, appreciate that many changes can be made in the specific embodiments which are disclosed and still obtain a like or similar result without departing from the spirit and scope of the invention.

EXAMPLE 1

Protein Absorption in the Intestinal Walls

[0037] Both human and animal models were used for measuring protein absorption in the intestinal walls. The subjects were prepared with indwelling catheters to enable blood sampling. Stable isotopes of amino acids are obtained and made into sterile and pyrogen-free solutions. These isotopes may comprise L-allo-[$^{2}H_{10}$, ^{15}N]-isoleucine, L-allo-[$^{13}C_{6}$]-isoleucine, D-1-[^{13}C]phenylalanine, D-C$^{3}C_{6}$] phenylalanine, D-[$^{2}H_{5}$]-phenylalanine, D-[2-^{2}H]phenylalanine. A blood sample is taken or drawn from each subject for a measurement of the natural background. A bolus or continuous infusion of the stable isotope of the amino acid may be used. The labeled amino acid is administered orally to each subject and another isotope of the amino acid nutrient is administered intravenously to the same subject at the same time or within **8** hours. Blood samples are taken or drawn from each subject in the time window of about one to several hours. The labeled amino acids enrichments in each sample are analyzed by Mass Spectrometry. Results from the Mass Spectrometry are applied to a non-compartmental model for analysis and ratio determination. The metabolic phenotype is produced based on the measured amino acids production, consumption and metabolism.

[0038] The results in FIG. **1** show that the absorption ratio of orally provided L-allo-isoleucine isotopologue over intravenously provided L-allo-isoleucine isotopologue is about 0.8 in healthy volunteers, which is in line with the expectation. FIG. **2** shows the ratio of orally provided D-phenylalanine isotopologue over intravenously provided D-phenylalanine isotopologue in healthy volunteers. FIG. **3** shows that the absorption ratio of D-phenylalanine isotopologues correlates with the absorption ratio of L-allo-isoleucine isotopologues with a p value less than 0.0001.

[0039] For pig models, the results are shown in FIGS. **4** and **5**. FIG. **4** shows that for continuously fed pig (4 hours), the ratio of enterally administered L-allo-[$^{13}C_{6}$]-isoleucine over intravenously administered L-allo-[$^{2}H_{10}$, ^{15}M-isoleucine is about 0.8. FIG. **5** shows the result for pigs with bolus feeding for 4 hours. The ratio of enterally administered allo-isoleucine[$^{13}C_{6}$](M6) over intravenously administered allo-Isoleucine[$^{2}H_{10}$, ^{15}N](M9+M11) is also about 0.8. However, FIG. **5** shows greater oscillation of the ratios than FIG. **4**.

EXAMPLE 2

Measurement for Other Nutrients

[0040] Protein and other macro-nutrients are tested in both animal and human models with both healthy and absorption impaired subjects. A similar method was used to measure fat

and carbohydrate absorption. To measure monosaccharide absorption, absorption isotopologue combinations of 3-O-Methylhistidine are used. For measurement of fat, absorption isotopologues of heptadecanoic acid are used.

EXAMPLE 3

Protein Digestion and Absorption in Chronic Heart Failure (CHF) and Chronic Obstructive Pulmonary Disease (COPD) Patients

[0041] Protein digestion and absorption are measured simultaneously in CHF and

[0042] COPD patients and healthy control subjects. Digestion, absorption, splanchnic extraction and turnover of the key amino acid phenylalanine in human protein metabolism are measured using a combination of enteral or oral and intravenous stable isotopes such as phenylalanine and isoleucine. Stable isotope molecules are slightly heavier and therefore less abundant in nature than their less heavy counterparts. Differences in weight and labeling patterns enable identification and quantification of metabolic pathways.

Digestion

[0043] A ^{15}N-labeled spirulina protein, which contains a.o. L-[^{15}N]phenylalanine, is used with a differently labeled stable isotope of L-phenylalanine, for example, L-[$^{2}H_5$]phenylalanine or -[$^{13}C_6$]phenylalanine orally to enable measurement of digestibility (FIG. **6**). The enrichment ratio L-[^{15}N]phenylalanine/L-$^{2}H_5$]phenylalanine would be close to 1 if spirulina is fully digested, since L-[^{15}N]phenylalanine, which is released from spirulina, and free L-[$^{2}H_5$]phenylalanine are taken up at the same rate. It was previously determined that the digestibility of spirulina is 80% in healthy humans and the same observed digestibility is expected in healthy volunteers.

Absorption

[0044] L- and D-amino acids are both absorbed at the same rate, but only L-amino acids are used for protein synthesis. Absorbed D-phenylalanine is secreted in urine or oxidized by D-amino oxidase. D-phenylalanine is present in plasma at levels, comparable to the levels reached with infusion of the D-phenylalanine stable isotopes. Because orally ingested D-phenylalanine will escape gut and liver metabolism, a combination of orally ingested and intravenously administered labeled D-phenylalanine, such as D-1-^{13}C-phenylalanine and [ring-$^{2}H_5$]phenylalanine, are used to measure absorption. The ratio between the D-phenylalanine stable isotope enrichments is close to 1 when absorption is unimpaired. A combination of stable isotopes of L-allo-isoleucine orally and intravenously, such as L-allo-[$^{13}C_6$]isoleucine and L-allo-[$^{2}H_{10}$, ^{15}N]isoleucine, are used to measure absorption. L-allo-isoleucine also escapes gut and liver metabolism, because it cannot be used for protein synthesis, and is slowly catabolized by the human body due to competition from other branched chain amino acids. Therefore, the ratio between enrichments of L-allo-$C^{3}C_6$]isoleucine and L-allo-[$^{2}H_{10}$, ^{15}N]isoleucine in plasma are close to **1** when absorption of amino acids is not impaired. Although formation of L-allo-isoleucine is a common metabolic event in humans, its concentration in blood is low and is in the range of that reached with the infusions of the L-allo-isoleucine stable isotopes. D-Amino acids and allo-isoleucine are also present in food. There are no negative effects reported from these metabolites in naturally occurring amounts in food and the human body. Moreover, the amount of stable isotopes provided herein is very low in comparison to naturally present concentrations of phenylalanine and allo-isoleucine in food.

Splanchnic Extraction

[0045] Splanchnic extraction is measured using the ratio between the already orally provided stable isotope of L-phenylalanine for measurement of digestion, for example L-[$^{3}C_6$]phenylalanine, and the intravenously provided stable isotope of phenylalanine with a different labeling pattern, such as L-[$^{13}C_9$]phenylalanine. Orally, **2** or **3** other labeled amino acids, such as L-[methyl-$^{3}H_2$]methionine and L-[3,3-$^{2}H_2$]tyrosine can be added, to establish digestion of these amino acids from spirulina in healthy people to examine the consequences of the delivery of undigested proteins and other nutrients to the large intestine in patients. Additionally, protein synthesis and breakdown is measured using an intravenously provided stable isotope of L-phenylalanine and L-tyrosine, such as L-[U-$^{13}C_9$-^{15}N]tyrosine, to correlate with total protein turnover.

Confirmation of Conversion Rate

[0046] Using only the D-phenylalanine and L-alloilsoleucine stable isotopes in the subjects enables confirmation in humans of a **10**% conversion rate of D- into L-phenylalanine that was observed in the pig. This allows for an estimation of the potential disturbance of the digestion and splanchnic extraction measurements that also involve L-phenylalanine stable isotopes.

Protocol

[0047] Body weight, height and vital signs will be assessed and body composition (muscle mass, fat mass and bone density) is determined by Dual Energy X-ray Absorptiometry (DXA) and/or Bioelectrical impedance analysis (BIA). As shown in FIG. **7**, after a background or baseline blood sample is taken, subjects receive a primed, continuous intravenous solution of stable isotopes, such as D-[ring-$^{2}H_5$]phenylalanine and L-[$^{13}C_9$]phenyalanine, L-allo-isoleucine, for example, L-allo-[$^{2}H_{10}$, ^{15}N]isoleucine, and, when protein turnover is measured, L-tyrosine, such as L-[U-$^{13}C_9$-^{15}N]tyrosine. Subsequently, stable isotope solutions are administered intravenously and with sip feeding. Subjects consume a liquid nutrition formula containing protein and carbohydrates in sips every 20 min thereby receiving approximately one-third of their total daily protein needs based on a requirement of ±1 g/kg/day. Stable isotopes diluted in the sip feeding are ^{15}N-labeled spirulina protein, which contains ^{15}N-phenyalanine), and stable isotopes of L-phenylalanine (L-Phe), such as L-[$^{13}C_6$]Phe, D-Phe, such as D-[1-^{13}C]Phe, and L-allo-isoleucine (L-allo-Ile), such as L-allo-[$^{13}C_6$]Ile. More stable amino acid isotopes such as stable isotopes of methionine (Met) and tyrosine (Tyr), for example, L-[methyl-$^{3}H_2$]Met and L-[3,3-$^{2}H_2$]Tyr, may be added. Arterialized-venous blood samples are taken throughout the study, before sip feedings, for analysis of stable isotope enrichments. The plasma or serum is separated from the blood and stored at −80° C. until analyses.

Enrichments in plasma will be measured using LC-MS/MS. At the 5th hour the subject receives a continuous sip feeding every 20 min for 3 hours.

EXAMPLE 4

Anabolic Effect of High-Quality Protein Sip Feeding in COPD, CHF and Cancer Patients

[0048] The dose-response anabolic effects of proteins with high EAA levels are examined in chronic heart failure (CHF), chronic obstructive pulmonary disease (COPD) and cancer patients. This aids in the determination in the actual protein requirements in these patients. This enables one to refine and personalize nutritional supplementation in COPD and CHF subjects to stop and even to restore muscle mass from progressive muscle wasting and to stop the ongoing muscle loss in cancer patients.

Protocol

[0049] Body weight, height, waist-hip ratio and vital signs are assessed and body composition (muscle mass, fat mass and bone density) is determined by bioelectrical impedance analysis (BIA), deuterium labeled water and/or Dual Energy X-ray Absorptiometry (DXA). As shown in FIG. **8** on every test day a background blood sample is collected. After the background blood samples are collected, subjects will receive IV pulse(s) containing stable isotopes such as L-[ring-$^{13}C_6$]-Phenylalanine, L-[ring-D_4]Tyrosine, L-[Methyl-D_3]Tau-Methylhistidine, trans-[2,5,5-D_3]4-Hydroxy-L-proline, L-[Guanido-$^{15}N_2$]-Arginine, L-[4,4,5,5-D_4-5-^{13}C]Citruline, L-$^{13}C_5$ -Ornithine, L-$^{15}N_2$-Glutamine, L-[1,2-$^{13}C_2$]Glutamic Acid, 1-^{13}C-Glycine, L-$^{15}N_3$-Histidine, L-$^{13}C_6$-Leucine, L-[methyl-D_3]Methionine, α-1-^{13}C-ketoisocaproic acid, L-1-^{13}C-Methionine, DL-[3,3,4,4-D_4] Homocysteine, L[1,2-$^{13}C_2$]Taurine, L-(Indole-D_5) Tryptophan, ^{13}C-Urea, L-1-^{13}C-Isoleucine, L-$^{13}C_5$-Valine, α-[Dimethyl-$^{13}C_2$]ketoisovaleric acid, 2-keto-3-methyl-$^{13}C_6$-pentanoate, 3-hydroxy-[3,4-methyl-$^{13}C_3$]isovaleric acid, $^{13}C_3$-glycerol.

[0050] After 2 hours, subjects receive stable isotopes by IV infusion, i.e., primed continuous, such as L-[ring-$^2H_5$]-phenylalanine, L-[$^{13}C_9$, ^{15}N]-tyrosine, 1-^{13}C-Tryptophan, a pulse, such as L-[allo-D_{10}-^{15}M-isoleucine, or by oral intake, such as L-[allo-$^{13}C_6$]-isoleucine. The subjects also drink the loading dose of D_2O tracer. After 4 hours, the subjects receive a high-quality protein meal as a sip feeding every 20 minutes for next 4 hours. Each sip feeding contains a mixture of casein hydrolysate with additional carbohydrates and may contain oral stable isotopes, such as 1-^{13}C-phenylalanine, [^{15}N]-spirulina, to measure the anabolic response to feeding and to protein digestion rates.

[0051] Subjects complete questionnaires to measure quality of life, physical activity, mood and depression and complete simple neuropsychological tests to assess cognition. Resting energy expenditure, if applied, is measured by ventilated hood technique between the 2nd and 3th hour of the test day. Handgrip dynamometry and a one leg exercise test using a short protocol on the Kin Com device may be used to assess hand and leg muscle strength and endurance. Inspiratory and expiratory muscle strength is assessed by using a hand-held mouth pressure device.

EXAMPLE 5

Anabolic Response to Feeding and Protein Digestion in Pre- and Post-Bariatric Surgery Patients

[0052] The variation in % weight loss due to the very low calorie diet followed by obese subjects prior to bariatric surgery and in percent weight loss 3 months after bariatric surgery is examined with regard to baseline genomic markers, gene expression profile, proteomic and metabolomic signatures as well as baseline metabolic and substrates fluxomics response to a defined meal.

Protocol

[0053] The protocol in Example 4 is followed as shown in FIG. **8**. In addition on the first test day a blood sample is drawn for genetic analysis.

EXAMPLE 6

Effect of Dietary Whey Protein on Serotonin Metabolism and Learning and Memory Functions in Older Adults

[0054] The effect of an acute intake of whey protein fraction with high levels of tryptophan precursor (TRP), such as alpha-lactalbumin, on the stabilization of the metabolism of serotonin metabolism and subsequent enhancement of metabolic and cognitive functions in older adults is examined.

Protocol

[0055] Body weight, height and vital signs will be assessed and body composition (muscle mass, fat mass and bone density) is determined by Dual Energy X-ray Absorptiometry (DXA) and/or Bioelectrical impedance analysis (BIA). Before administration of the IV continuous infusion and pulse, venous blood is collected for measurement of the natural enrichment of amino acids, CRP, urea, cortisol, glucose, insulin, inflammatory mediators, blood lipids, bone panel. Administration of the stable isotopes, such as L-[ring-$^2H_5$]-phenylalanine, L-[$^{13}C_9$, ^{15}N]-tyrosine, and D5-tryptophan, starts through constant continuous infusion to measure protein kinetics. A pulse of stable isotopes such as $^{15}N_2$, TRP, $^2H_3$-Leucine, L-[$^2H_3$]-3-Methylhistidine, $^2H_2$-Glycine, 2-D-hydroxyproline, $^{1\text{-}13}$C-ketoisocaproic acid (KIC), L-[Guanido-$^{15}N_2$]-Arginine, L-[ureido-^{13}C-$^2H_2$]-Citrulline), L-Glutamine-amide-^{15}N, 1,2-$^{13}C_2$-L-Glutamic acid, 1,2-13C2Taurine, and 13C-Urea is given IV simultaneously to measure tryptophan and leucine turnover, myofbrillar protein breakdown (reflecting muscle protein breakdown), glutathione turnover, hydroxyproline breakdown (reflecting collagen breakdown), glutamine turnover, glutamate turnover, taurine turnover, and urea production. L-[$^2H_4$]-tyrosine and the other tracers will be primed IV and/or orally if needed. Around 3 hours after the start of study, subjects orally ingest a protein supplement to which stable isotopes such as L-[$^{1\text{-}13}$C]-phenylalanine and ^{15}N-spirulina are added to the nutritional supplement to be able to measure splanchnic extraction of the dietary amino acid and digestion rates of protein (purchased from Cambridge Isotopic Laboratories, Woburn, Mass. or Sigma-Aldrich, St. Louis, Mo.). On all test days, during IV infusion, subjects complete a list of questions regarding quality of life, mood and depression, physical activity level and to complete simple neuropsychological tests to assess cognition.

EXAMPLE 7

Fat Metabolism in Obstructive Sleep Apnea and Chronic Obstructive Pulmonary Disease Patients

[0056] De novo lipogenesis, triglyceride synthesis and breakdown, and fatty acid oxidation in the liver and adipose tissue and digestion, whole body protein and glucose kinetics are examined in OSA and COPD patients. Protein, carbohydrate, and fat metabolism are examined in healthy subjects as a control.

Protocol

[0057] Body weight, height and vital signs will be assessed and body composition (muscle mass, fat mass and bone density) is determined by Dual Energy X-ray Absorptiometry (DXA) and/or Bioelectrical impedance analysis (BIA). Before administration of the drink containing stable isotopes, venous blood will be collected for measurement of the natural enrichment of plasma water and metabolites. On test day 1, a background urine and blood sample will be collected. Subjects then receive the stable isotope by pulse, continuous infusion and/or by ingestion. Early on test day 2, a blood sample is collected and then stable isotopes, such as glycerol, tyrosine, phenylalanine, glucose, arginine, and citrulline, are administered intravenously, by a pulse and/or by ingestion. On test day 2, a test meal is or is not provided and may contain stable isotopes (purchased from Cambridge Isotopic Laboratories, Woburn, Mass. or Sigma-Aldrich, St. Louis, Mo.). A subcutaneous adipose tissue biopsy may or may not be collected from the abdomen. Following the biopsy, more stable isotopes may be administered intravenously. After the meal an additional adipose tissue biopsy can be collected to determine adipose tissue de novo lipogenesis and triglyceride synthesis.

[0058] The present invention is well adapted to attain the ends and advantages mentioned as well as those that are inherent therein. The particular embodiments disclosed above are illustrative only, as the present invention may be modified and practiced in different but equivalent manners apparent to those skilled in the art having the benefit of the teachings herein. Furthermore, no limitations are intended to the details of construction or design herein shown, other than as described in the claims below. It is therefore evident that the particular illustrative embodiments disclosed above may be altered or modified and all such variations are considered within the scope and spirit of the present invention. Also, the terms in the claims have their plain, ordinary meaning unless otherwise explicitly and clearly defined by the patentee.

What is claimed is:

1. A method for diagnosing impaired absorption of a nutrient in an individual, comprising the steps of:

administering a first stable absorption isotopologue of said nutrient orally to the individual;

administering a second stable absorption isotopologue of the same nutrient intravenously to said individual;

drawing blood samples periodically from said individual;

measuring an amount of the first isotopologue and of the second isotopologue in each sample;

calculating a ratio of the first isotoologue to the second isotopologue in each sample; and

comparing the ratio calculated for the individual to a ratio calculated for a healthy control; wherein, when the ratio calculated for the individual is less than the ratio for the healthy control, impaired absorption of the nutrient in said individual is present.

2. The method of claim **1**, further comprising:

analyzing the nutrient production, consumption and metabolism; and

producing a metabolic phenotype based on the analysis.

3. The method of claim **2**, wherein the individual has chronic heart failure, chronic obstructive pulmonary disease, a cancer, a cognitive dysfunction, or is a bariatric pre-operative or post-operative patient.

4. The method of claim **1**, wherein the ratio of impaired absorption is **1.0** or less in the individual.

5. The method of claim **1**, wherein the first labeled nutrient and the second labeled nutrient are administered simultaneously or said second labeled nutrient is administered within **8** hours of said first labeled nutrient.

6. The method of claim **1**, wherein an isotope comprising the isotopologue is ^{2}H, ^{13}C, or ^{15}N.

7. The method of claim **1**, wherein said nutrient is an amino acid, carbohydrate, fat, or a combination thereof that is not metabolized in the gut of the individual.

8. The method of claim **7**, wherein the isotopologue of the amino acid comprise L-Allo-[$^{2}H_{10}$, ^{15}M-Isoleucine, L-Allo-[$^{13}C_6$]-Isoleucine, D-1-[^{13}C]phenylalanine, D-[$^{13}C_6$]phenylalanine, D-[$^{2}H_5$]-phenylalanine, D-[2-^{2}H]phenylalanine or a combination thereof.

9. The method of claim **7**, wherein the isotopologues of carbohydrates comprise an isotope of 3-O-methylglucose or an isotope of non-metabolized analogues thereof or a combination thereof.

10. The method of claim **7**, wherein the isotopologues of fat comprise an isotope of heptadecanoic acid an isotope of non-metabolized analogues thereof or a combination thereof.

11. The method of claim **1**, wherein the calculating step comprises applying a non-compartmental model to the measured amounts of the first isotopologue and the second isotopologue.

12. A method for determining an intestinal absorption level of a nutrient in an individual, comprising the steps of:

administering a first stable absorption isotopologue of the nutrient enterally to the individual;

administering a second stable absorption isotopologue of the same nutrient intravenously to the patient;

drawing blood samples periodically from said patient;

measuring an amount of the first isotopologue and of the second isotopologue in each sample; and

calculating a ratio of the first isotopologue to the second isotopologue in each sample, said ratio correlating to the intestinal absorption level in the individual.

13. The method of claim **12**, further comprising:

calculating a ratio of the first isotopologue to the second isotopologue after administration to a healthy control; and

comparing the ratio calculated for the individual to the ratio calculated for the healthy control; wherein a decrease in the ratio for the individual compared to the ratio for the healthy control comprises a diagnosis of impaired absorption of the nutrient in said individual.

14. The method of claim **13**, wherein the ratio in the individual is **1.0** or less.

15. The method of claim **12**, wherein the first labeled nutrient and the second labeled nutrient are administered

simultaneously or said second labeled nutrient is administered within **8** hours of said first labeled nutrient.

16. The method of claim **12**, wherein an isotope comprising the isotopologue is ^{2}H, ^{13}C, or ^{15}N.

17. The method of claim **11**, wherein said nutrient comprises a non-metab amino acids, carbohydrates, fat or a combination thereof, said nutrient not metabolized in the gut of the individual.

18. The method of claim **17**, wherein the isotopologue combinations of amino acids comprise L-Allo-[$^{2}H_{10}$, ^{15}M-Isoleucine, L-Allo-[$^{13}C_{6}$]-Isoleucine, D-1-[^{13}C]phenylalanine, D-C$^{3}C_{6}$]phenylalanine, D-[$^{2}H_{5}$]-phenylalanine, D-[2-^{2}H]phenylalanine or a combination thereof.

19. The method of claim **17**, wherein the isotopologue combinations of carbohydrates comprise an isotope of 3-O-methylglucose or any other non-metabolized analogues of carbohydrates or a combination thereof.

20. The method of claim **17**, wherein the isotopologue combinations of fat comprise an isotope of heptadecanoic acid or any other non-metabolized analogues of fatty acids or a combination thereof.

21. The method of claim **12**, wherein the calculating step comprises applying a non-compartmental model to the measured amounts of the first isotopologue and the second isotopologue.

* * * * *
