# Supplementary material for: Goat milk protein digestibility in relation to intestinal function
Source: Am J Clin Nutr. 2021 Mar 1;113(4):845–53. doi: 10.1093/ajcn/nqaa400 (PMC8023838; doi:10.1093/ajcn/nqaa400)
Supplement: nqaa400_Supplemental_Files [file nqaa400_supplemental_files.zip › Online Supplementary Material.docx]

Supplementary Figure 1. Subject screening and enrollment details

Assessed for eligibility (*n*=9)

Excluded (*n*=2)

Not meeting inclusion criteria

Dual sugar assay (*n*=7)

Milk digestibility and allo-isoleucine absorption index studies in randomized order (*n*=7)

Excluded (*n*=1)

Participant met with an accident

All subjects completed the study and were included for data analysis

Milk digestibility study (*n*=7)

Allo-isoleucine absorption index (*n*=6)

Supplementary Figure 2. ^2^H indispensible amino acid enrichments of intrinsically labelled maize and cowpea fodder expressed as parts per million excess (ppme)

Supplementary Table 1. Ingredients and nutrient composition of protein-free wheat starch cookies^1^.

| Ingredients | Amount  (g) | Carbohydrate (g) | Protein  (g) | Fat  (g) | Energy (kcal) |
| --- | --- | --- | --- | --- | --- |
| Protein free wheat starch | 156.0 | 134.3 | 0.3 | 0.1 | 541.3 |
| Butter | 86.0 | 0.1 | 0.0 | 69.8 | 616.6 |
| Vanilla flavour | 1.2 | 0.0 | 0.0 | 0.0 | 0.0 |
| Baking powder | 2.0 | 0.7 | 0.0 | 0.0 | 2.7 |
| Beet sugar | 38.4 | 37.7 | 0.0 | 0.0 | 150.9 |
| Salt | 0.5 | 0.0 | 0.0 | 0.0 | 0.0 |
| Oil | 5.4 | 0.0 | 0.0 | 5.4 | 48.6 |
| Total | 289.5 | 172.7 | 0.3 | 75.2 | 1360.2 |

^1^The ingredients represented in the table is required for preparation of six cookies.

Supplementary Table 2. Dynamic MRM transitions monitored in allo-isoleucine absorption index study.

| Compound Name | Precursor Ion | Product Ion |
| --- | --- | --- |
| Allo-Ileu ^13^C_6_ ^15^N_1_ | 239.15 | 164.12 |
| Allo-Ileu ^13^C_6_ | 238.15 | 163.12 |
| Allo-Ileu ^13^C_5_ | 237.15 | 163.12 |
| Allo-Ileu ^2^H_10_ | 242.15 | 168.12 |
| Allo-Ileu ^2^H_9_ | 241.15 | 167.12 |
| Allo-Ileu ^2^H_8_ | 240.15 | 166.12 |

Supplementary Table 3. Mean enrichments of ^2^H and ^13^C labelled indispensible amino acids in test meal administered to apparently healthy South Indian women in milk digestibility study expressed as parts per million excess (ppme).^1^

| Amino Acids | ^2^H | ^13^C |
| --- | --- | --- |
| Methionine | 206.7 ± 8.0 | 2621.0 ± 226.3 |
| Phenylalanine | 306.6 ± 9.0 | 1749.1 ± 31.2 |
| Threonine | 230.7 ± 18.0 | 2375.4 ± 166.0 |
| Lysine | 164.1 ± 5.9 | 1782.0 ± 181.2 |
| Leucine | 281.2 ± 5.2 | 4859.9 ± 670.5 |
| Iso-leucine | 230.9 ± 17.0 | 4955.8 ± 819.5 |
| Valine | 253.5 ± 10.5 | 4440.4 ± 716.6 |

^1^Values are mean ± SD
